# The checkpoint inhibitor PD-1H/VISTA controls osteoclast-mediated multiple myeloma bone disease

**DOI:** 10.1038/s41467-023-39769-8

**Published:** 2023-07-17

**Authors:** Jing Fu, Shirong Li, Huihui Ma, Jun Yang, Gabriel M. Pagnotti, Lewis M. Brown, Stephen J. Weiss, Markus Y. Mapara, Suzanne Lentzsch

**Affiliations:** 1 Columbia University Irving Medical Center, Department of Medicine, New York, NY USA; 2 Columbia Center for Translational Immunology, New York, NY USA; 3 Indiana University, Indianapolis, IN USA; 4 University of Texas—MD Anderson Cancer Center, Houston, TX USA; 5 Quantitative Proteomics and Metabolomics Center, Columbia University, New York, NY USA; 6 Department of Internal Medicine, Life Sciences Institute, University of Michigan, Ann Arbor, MI USA

**Keywords:** Cancer microenvironment, Myeloma, Mechanisms of disease

## Abstract

Multiple myeloma bone disease is characterized by the development of osteolytic bone lesions. Recent work identified matrix metalloproteinase 13 as a myeloma-derived fusogen that induces osteoclast activation independent of its proteolytic activity. We now identify programmed death-1 homolog, PD-1H, as the bona fide MMP-13 receptor on osteoclasts. Silencing PD-1H or using *Pd-1h*^*-/*-^ bone marrow cells abrogates the MMP-13-enhanced osteoclast fusion and bone-resorptive activity. Further, PD-1H interacts with the actin cytoskeleton and plays a necessary role in supporting c-Src activation and sealing zone formation. The critical role of PD-1H in myeloma lytic bone lesions was confirmed using a *Pd-1h*^*-/-*^ myeloma bone disease mouse model wherein myeloma cells injected into *Pd-1h*^*-/-*^*Rag2*^*-/-*^ results in attenuated bone destruction. Our findings identify a role of PD-1H in bone biology independent of its known immunoregulatory functions and suggest that targeting the MMP-13/PD-1H axis may represent a potential approach for the treatment of myeloma associated osteolysis.

## Abstract (web summary)

Multiple myeloma bone disease is characterized by the development of osteolytic bone lesions. Here, the authors demonstrate that the checkpoint inhibitor PD-1H functions as a potential MMP-13 receptor on osteoclasts, mediating MMP-13 induced cytoskeleton reorganization, fusogenesis and bone resorption and further regulating osteolytic lesions.

## Introduction

Multiple myeloma (MM) is the second most common hematologic malignancy with a characteristic development of pure lytic bone lesion in 80% of patients^1^. MM cells secrete pro-osteoclastogenic factors, which lead to osteoclast (OCL) activation and bone lesions, evidenced by the fact that MM cells are commonly found adjacent to active bone resorption sites^2^. Current treatments that target osteoclast function, including bisphosphonates and RANKL inhibitors, however, only partially inhibit osteolytic lesions and are accompanied by side effects such as osteonecrosis of the jaw (ONJ)^1,2^. As such, alternative therapies that specifically intercept the osteoclastogenic factors that are generated at the MM-OCL interface are needed to improve treatment outcome.

Recent work demonstrated that matrix metalloproteinase 13 (MMP-13) serves as a critical osteoclastogenic factor that is highly expressed by human MM cells, thereby mediating MM-induced bone lesions^3^. Interestingly, MMP-13 enhances RANKL-induced osteoclast multinucleation and bone-resorption by triggering the ERK1/2-depedent upregulation of the cell fusogen, dendritic cell-specific transmembrane protein (DC-STAMP), via a process that operates independently of the enzyme’s proteolytic activity. Further, in the 5TGM1 MM bone disease mouse model, silencing MMP-13 expression in myeloma cells inhibits the development of osteolytic lesions^3^.

The fact that MMP-13 induces OCL signaling independently of its proteolytic activity is consistent with a model wherein osteoclastogenic activity is mediated via heretofore uncharacterized ligand-receptor interactions. To this end, using mass-spectrometry to screen for potential MMP-13 receptors on OCLs, we now identify the checkpoint protein, programmed death-1 homolog (PD-1H), as a functional binding partner for the metalloenzyme. PD-1H co-localizes with OCL F-actin, and following MMP-13 binding, the OCL cytoskeleton undergoes reorganization, fusion is triggered and bone resorption activated in wild-type, but not *Pd-1h* knockout cells. Mechanistically, we find that MMP-13/PD-1H binding triggers c-Src/Rac1 activation that, in turn, regulates both OCL fusogenesis and sealing zone formation. Finally, we demonstrate that in a mouse model of MM bone disease, *Pd-1h* deficiency mitigates the formation of MM-induced bone lesions. Together, these findings identify the MMP-13/PD-1H axis as a key regulator —and potential point of therapeutic intervention—in MM-triggered osteolysis.

## Results

### Identification of PD-1H as an MMP-13 receptor

**Fig. 1:**
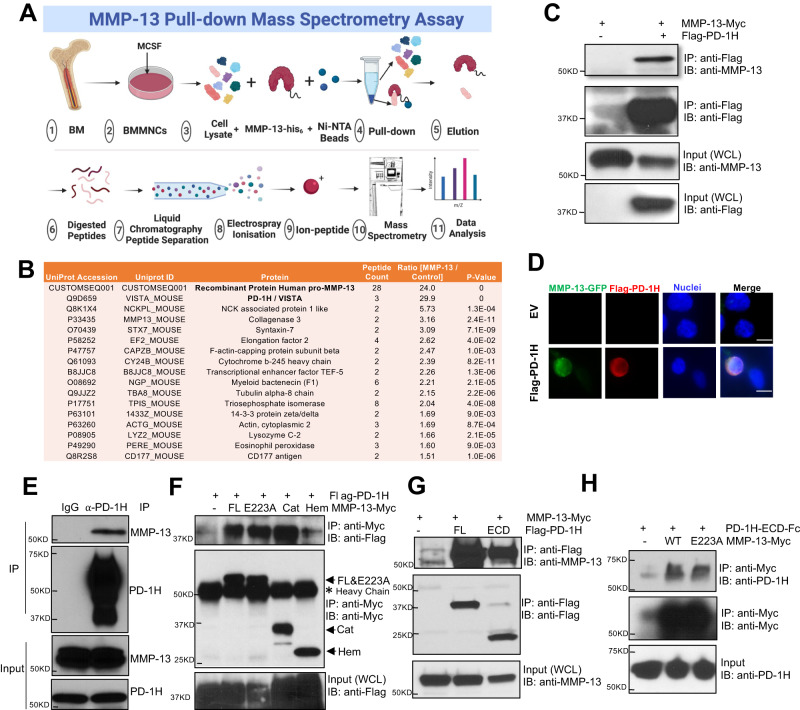
MMP-13 binds to PD-1H expressed on OCLs. **A** For MMP-13 pull-down, BSA or MMP-13-His_6_ recombinant protein were incubated with M-CSF primed mouse non-adherent mononuclear bone marrow cell lysates, followed by Ni-NTA agarose beads affinity pull-down. MMP-13 binding proteins were extracted and analyzed by LC/MS/MS Mass spectrum. Figure was created with BioRender.com. **B** Among all the proteins, PD-1H showed the highest relative abundance in the MMP-13 pull-down samples compared to BSA control samples (*n* = 3, each sample was analyzed twice). *P*-values were determined by two-sided Student’s *t*-test. **C** HEK293 cells (not expressing MMP-13) were transfected with Myc tagged MMP-13 alone or together with Flag-PD-1H, followed by IP using Flag antibody. Whole-cell lysates (WCL) were analyzed as input. The experiment was performed three times with similar observations. **D** HEK293 cells were transfected by empty vector (EV) or Flag-PD-1H, followed by incubation with MMP-13-GFP fusion protein. Cells were stained with FITC-anti-GFP, PE-anti-PD-1H and nuclei by DAPI. Scale bar, 10 μm. The experiment was performed three times with similar observations. **E** Mouse OCL cell lysates were incubated with RPMI8266 conditioned serum free medium, followed by IP using anti-PD-1H antibody. Rabbit IgG was used as control. The experiment was performed three times with similar observations. **F** HEK293 cells were transfected with Flag-PD-1H alone or together with Myc tagged full-length MMP-13 (FL), E223A enzymatic dead mutant, pro-catalytic domain (Cat, aa 1–267) or Hemopexin domain (Hem, deletion aa 37–267), followed by IP using anti-Myc (9E10) antibody. WCL were analyzed as input. The experiment was performed three times with similar observations. **G** HEK293 cells were transfected with MMP-13 alone or together with Flag-PD-1H full-length (FL) or extracellular domain (ECD), followed by IP using Flag antibody. Whole-cell lysates were analyzed as input. The experiment was performed three times with similar observations. **H** Purified PD-1H-ECD-Fc recombinant protein was incubated with purified MMP-13 WT-Myc or E223A-Myc recombinant protein and pulled down by Myc magnetic beads. The experiment was performed three times with similar observations.

MMP-13 binding proteins in OCL were first screened by pull-down assay where His_6_-tag- conjugated, full-length pro-MMP-13 or a BSA control protein were incubated with M-CSF-primed mouse bone marrow monocytes (BMMNCs) lysates. Binding proteins were captured on Ni-NTA agarose followed by mass spectrometry (Fig. 1A). Each treatment was performed in triplicate, and each replicate was analyzed in duplicate arrays. Among the proteins identified in MMP-13 complexes, PD-1H was the most abundant target identified (signal ratio of MMP-13 pull-down *vs* control: 29.9), and the only cell surface protein (Fig. 1B, Supplemental Data File 1). 28 peptides were identified from the bait pro-MMP13 protein by mass spectrometry. This included a peptide spanning the zymogen activation site (aa 96–109) (CGVPDVGEYNVFPR), confirming the zymogen form, instead of the activated form, of MMP-13 in the pull-down complex (Supplemental Data File 2). Binding between PD-1H and MMP-13 was confirmed by co-immunoprecipitation (co-IP) assay in PD-1H-transfected HEK 293 cells (Fig. 1C). Direct binding was likewise observed in an intact cell system following incubation of an MMP-13-GFP fusion protein with PD-1H-expressing, but not in control, HEK 293 cells (Fig. 1D). Endogenous MMP-13 and PD-1H binding was also confirmed in co-IP assays using mouse OCL lysates incubated with MMP-13 expressing human MM cell line RPMI8266 conditioned medium (Fig. 1E).

To identify key binding domains between PD-1H and MMP-13, a series of truncated deletion mutants were tested. The MMP-13 zymogen has a pro-peptide domain (aa 20–103) that maintains latency, a catalytic domain (aa 103–268) containing the catalytic zinc-binding motif, and a hinge linker region (aa 269–280) that connects the catalytic domain region with the C-terminal hemopexin domain (aa 281–471)^4^. MMP-13 enzymatic activity is not required for binding to PD-1H as demonstrated by the similar binding activity of PD-1H to pro-MMP-13 WT full-length (FL) and a catalytically inactive E223A point mutant^3^ (Fig. 1F). By contrast, while the MMP-13 pro-catalytic domain alone (Cat, i.e., aa 1–267) displayed a binding affinity to PD-1H that was similar to full-length MMP-13, a truncation mutant containing the hemopexin domain (Hem, i.e., Δaa 37–267) retained only a fraction of the wild-type binding activity.

PD-1H is a transmembrane protein with N-terminal extracellular domain (aa 30–190), a single-pass transmembrane domain (aa 193–213) and a C-terminal intracellular domain (aa 214–310)^5,6^. As expected, in co-IP assays only the extracellular domain (ECD) of PD-1H bound to MMP-13 when co-expressed in HEK 293 cells, although binding activity was somewhat weaker than that of full-length (FL) PD-1H (Fig. 1G). To confirm the direct binding between PD-1H ECD and MMP-13, we incubated a purified MMP-13-Myc recombinant protein with a recombinant PD-1H ECD-Fc protein, and performed a pull-down assay using Myc-tag magnetic beads. Under these conditions, PD-1H ECD-Fc directly bound to MMP-13 (Fig. 1H). Consistent with the lack of any requirement for MMP-13 proteolytic activity, MMP-13 WT and MMP-13 E223A displayed similar binding to PD-1H ECD-Fc (Fig. 1H).

### PD-1H deficiency impairs MMP-13-induced OCL fusion

**Fig. 2:**
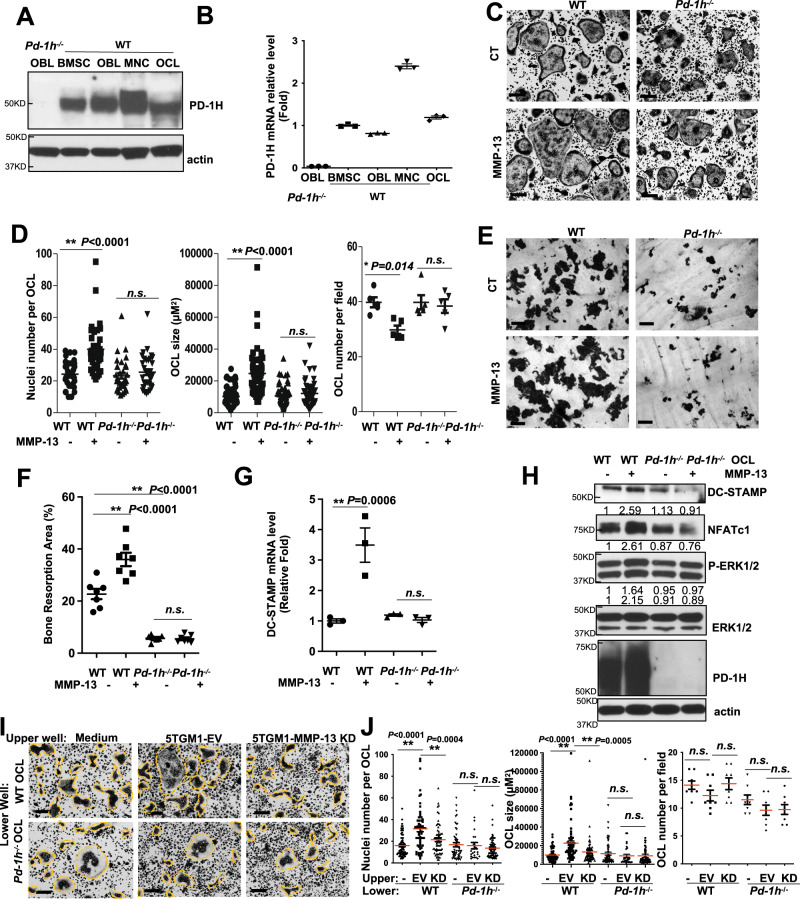
PD-1H deficiency impairs OCL fusion and bone resorption activity and blocks MMP-13 effects on OCL. **A**, **B** WT and *Pd-1h*^*-/-*^ BMCs were cultured for stromal cells, osteoblast, and osteoclast and PD-1H expression was analyzed by WB (**A**) and real-time PCR (**B**). Mean ± SD, *n* = 3 samples (**B**). **C**, **D** WT or *Pd-1h*^*-/-*^ mice BMCs were cultured for OCL formation ± MMP-13 followed by TRAP staining. Nuclei number per OCL, OCL size and OCL number per field were assessed from the randomly imaged fields of OCLs and analyzed by ImageJ. Scale bar, 100 μm (**C**). Nuclei number per OCL: *n* = 34 cells/group. OCL size: WT control *n* = 57 cells; WT + MMP-13 *n* = 52 cells; *Pd-1h*^*-/-*^ control *n* = 56 cells; *Pd-1h*^*-/-*^ + MMP-13 *n* = 56 cells. OCL number per field: *n* = 5 random fields. Mean ± SEM. *P ≤ 0.05, **P ≤ 0.01, by one-way ANOVA (**D**). **E**, **F** WT or *Pd-1h*^*-/-*^ OCLs were cultured atop bovine femur bone slices ± MMP-13 followed by hematoxylin staining. Bone resorption area (%) was calculated on randomly imaged fields (*n* = 7 fields) by ImageJ. Scale bar, 100 μm (**E**). Mean ± SEM. **P ≤ 0.01 by one-way ANOVA (**F**). **G**, **H** WT or *Pd-1h*^*-/-*^ BMCs were cultured for OCL ± MMP-13. DC-STAMP mRNA was detected by real-time PCR (*n* = 3 samples). Mean ± SEM. **P ≤ 0.01 by one-way ANOVA (**G**). Cell lysates were analyzed by WB (**H**). Bands densitometry intensities from repeated experiments are shown. The experiment was performed three times with similar observations. **I**, **J** WT or *Pd-1h*^*-/-*^ BMMNCs were co-cultured in transwell plates with either medium only, 5TGM1-EV or MMP-13 KD cells for OCLs followed by TRAP staining. Scale bar, 100 μm (**I**). Nuclei number per OCL, OCL size, and OCL number per field were determined as above. Nuclei number per OCL: WT MNC control *n* = 73 cells; WT MNC + 5TGM1 EV *n* = 59 cells; WT MNC + 5TGM1 KD *n* = 62 cells; *Pd-1h*^*-/-*^ MNC control *n* = 52 cells; *Pd-1h*^*-/-*^ MNC + 5TGM1 EV *n* = 25 cells; *Pd-1h*^*-/-*^ MNC + 5TGM1 KD *n* = 67 cells. OCL size: WT MNC control *n* = 70 cells; WT MNC + 5TGM1 EV *n* = 68 cells; WT MNC + 5TGM1 KD *n* = 64 cells; *Pd-1h*^*-/-*^ MNC control *n* = 58 cells; *Pd-1h*^*-/-*^ MNC + 5TGM1 EV *n* = 29 cells; *Pd-1h*^*-/-*^ MNC + 5TGM1 KD *n* = 65 cells. OCL number per field: *n* = 8 random fields. Mean ± SEM. *P ≤ 0.05, **P ≤ 0.01 by one-way ANOVA (**J**).

Consistent with a functional role for PD-1H in bone, PD-1H expression was detected in mouse bone marrow stromal cells (BMSCs), osteoblasts (OBLs), mononuclear cells (MNCs) and OCLs by western blotting and real-time PCR (Fig. 2A, B). PD-1H showed higher expression in MNCs compared to mature OCLs (Fig. 2A, B). To assess the role of PD-1H in supporting MMP-13-induced OCL fusion, PD-1H expression was silenced in mouse MNCs with a shRNA-PD-1H pGreenpuro lentiviral construct followed by GFP-based flow cytometry sorting^3,7^. PD-1H knockdown was confirmed by comparing empty vector (EV)- and sh-PD-1H knockdown (KD)-infected MNCs by quantitative real-time PCR and Western blotting (Supplemental Fig. 1A, B). EV and PD-1H KD MNCs were then cultured to induce OCL differentiation in the absence or presence of MMP-13^3^. Whereas MMP-13 successfully induced OCL fusion in EV MNCs, reflected by a significant increase in OCL size and nuclei number per OCL, PD-1H KD MNCs failed to respond (Supplemental Fig. 1C). In accordance with our prior findings, MMP-13 did not affect the total number of OCLs (Supplemental Fig. 1D). The purity of the infected cells was confirmed by GFP imaging as was the knockdown of PD-1H by PE-anti-PD-1H immunofluorescence (Supplemental Fig. 1E).

To further confirm the role of PD-1H in MMP-13-induced OCL fusion, OCL differentiation was also assessed using bone marrow MNCs isolated from *Pd-1h*^*-/-*^ mice and wild type (WT) littermates in the absence or presence of MMP-13. Consistent with the results obtained with PD-1H KD cells, MMP-13 failed to enhance OCL fusion in *Pd-1h*^*-/-*^ OCLs relative to WT controls (Fig. 2C, D). Even more strikingly, MMP-13-induced bone resorption activity was significantly impaired in *Pd-1h*^*-/-*^ OCLs when compared to WT OCLs (Fig. 2E, F).

Previous studies demonstrated that MMP-13 induces DC-STAMP expression in OCLs^3^. Accordingly, MMP-13 induced *Dc-stamp* mRNA upregulation^3^ (Fig. 2G), ERK signaling activation, NFATc1 and DC-STAMP upregulation at the protein level in WT, but not *Pd-1h*^*-/-*^ OCLs (Fig. 2H). We further assessed the role of PD-1H in MM cell-induced OCL formation. In MM-OCL co-culture assays performed in the absence of direct cell contact^3^, 5TGM1 MM cells (upper wells) induced the formation of large WT OCL from WT MNCs (lower wells). Knockdown of MMP-13 in 5TGM1 MM cells decreased OCL formation, confirming that this response was largely mediated by soluble MMP-13. However, when using *Pd-1h*^*-/-*^ MNCs for OCL formation, MMP-13 was unable to trigger effects on OCL fusion (Fig. 2I, J). Taken together, these data indicate that PD-1H expression in OCL precursor cell populations is critical for MM-induced, MMP-13-dependent OCL activation.

The role of MMP-13/PD-1H interaction was also verified in human osteoclasts. For this purpose, human cord blood CD34^+^ cells were transduced with pGreenpuro sh-h-PD-1H lentivirus followed by GFP^+^ cell sorting. PD-1H silencing in CD34^+^ cells and mature OCL was confirmed by western blotting (Supplemental Fig. 2A, B). Transduced CD34^+^ cells were then differentiated into OCLs in the absence or presence of MMP-13. As expected, in PD-1H-expressing cells, MMP-13 induced human OCL fusion as demonstrated by the increased nuclei number per OCL as well as OCL size in empty vector (EV) transduced, but not PD-1H knockdown, OCLs (Supplemental Fig. 2C, D).

### PD-1H is critical for F-actin cytoskeleton reorganization in OCLs

**Fig. 3:**
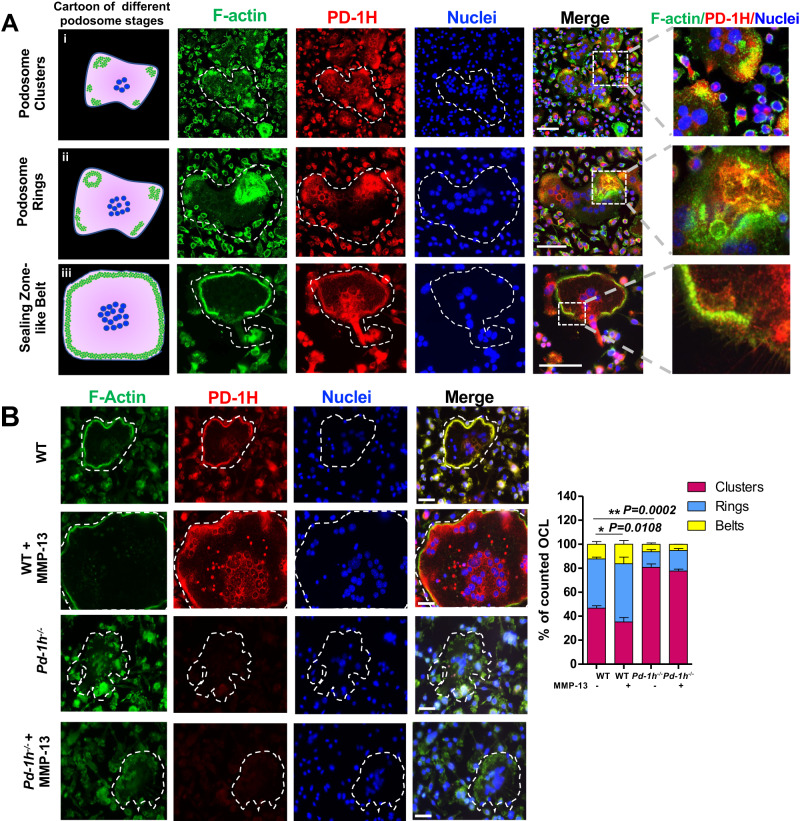
PD-1H co-localizes with podosome in OCL. **A** WT OCLs were stained with Acti-stain 488 phalloidin (for F-actin) (green), PE-anti-PD-1H (red) and DAPI (blue) and subjected to confocal microscopy. Left panel: cartoon of podosome (green) patterns during the different stages of OCL development. From top to bottom: clusters, rings, and sealing zone-like belt. Scale bar, 100 μm. The experiment was performed three times with similar observations. **B** BMCs from WT or *Pd-1h*^*-/-*^ mice were cultured in OCL differentiation medium without or with MMP-13 stained with Acti-stain 488 phalloidin (for F-actin) (green), PE-anti-PD-1H (red) and DAPI (blue). Podosome clusters, ring or sealing zone-like belt-forming OCL numbers per field were quantified from randomly imaged fields (*n* = 6/group). Mean ± SD. *P ≤ 0.05, **P ≤ 0.01 by one-way ANOVA. Scale bar, 50 μm.

Since *Pd-1h* knockout significantly decreased OCL-mediated bone resorption, we next addressed the role of PD-1H in cytoskeleton formation. The F-actin cytoskeleton is highly dynamic and undergoes unique patterns of reorganization critical for OCL differentiation and bone resorption activity^8,9^. Initially OCLs form F-actin-rich adhesive structures on the cell membrane, termed podosomes^8,10^. During later stages podosomes are collectively arranged into clusters and rings, and then finally, into either sealing zones (SZ) atop bone surfaces or SZ-like structures (also called belts) on non-mineralized substrates to mediate spreading, migration, and bone resorption^9,10^. Confocal microscopy demonstrated that PD-1H co-localizes with F-actin clusters (Fig. 3A panel i), rings (Fig. 3A panel ii) and sealing zone-like belts (Fig. 3A panel iii) as well as F-actin-associated proteins, such as cortactin^11^ (Supplemental Fig. 3A) and vinculin^12^ (Supplemental Fig. 3B) during podosome maturation. Vice versa, the disruption of F-actin filaments by the tropomyosin inhibitor, TR-100^13^, abrogated PD-1H co-localization with F-actin and accumulation in OCL sealing zones (Supplemental Fig. 4). Interestingly, 3D confocal microscopy reconstruction revealed that PD-1H is localized atop F-actin clusters/rings in WT OCL (Supplemental Fig. 5). By contrast, *Pd-1h* knockout leads to the disruption of podosome clusters at early stages relative to WT controls (Supplemental Fig. 5), while at later stages, *Pd-1h*^*-/-*^ OCLs exhibit significantly fewer F-actin rings and belts (Fig. 3B). As expected, MMP-13 did not trigger F-actin reorganization in *Pd-1h*^*-/-*^ OCLs, whereas in WT cultures, MMP-13 increased the number of OCLs forming F-actin rings and belts, as well as the size of F-actin belts (Fig. 3B). Interestingly, following the addition of MMP-13, we also observed an increased formation of zipper-like F-actin structures^14^ that facilitate OCL fusion, with PD-1H localized to the F-actin superstructure (Supplemental Fig. 6, panel ii).

### PD-1H mediates MMP-13-induced signaling of OCL cytoskeleton reorganization

**Fig. 4:**
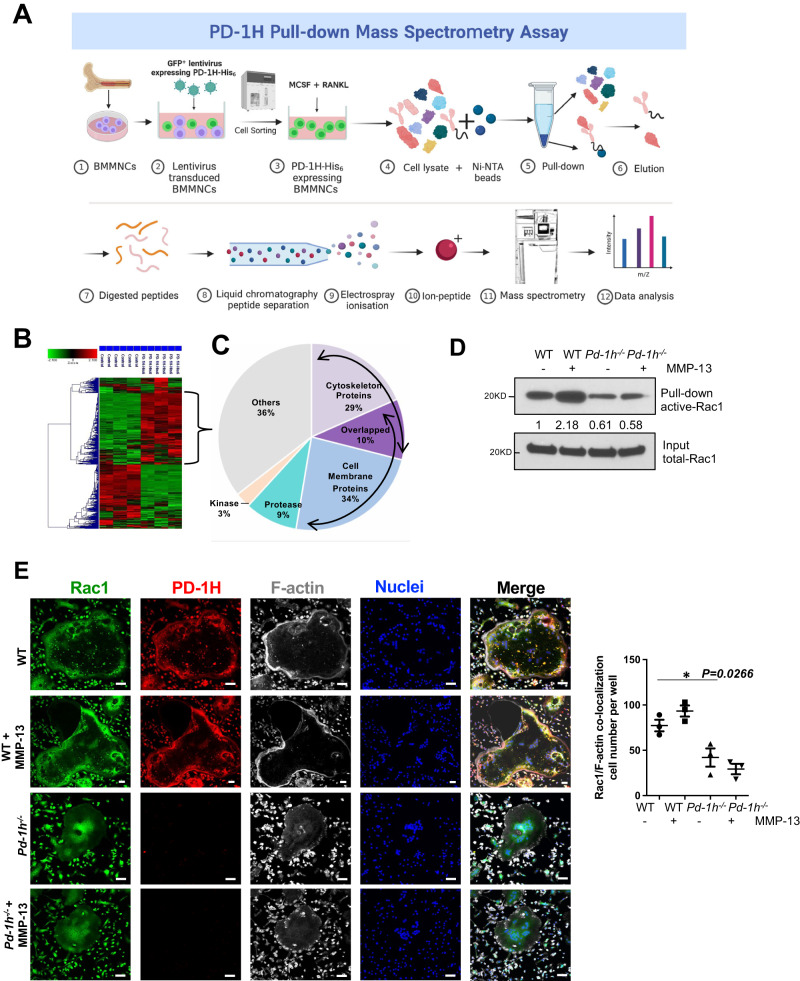
MMP-13 induces OCL formation via PD-1H binding to cytoskeleton proteins. **A**, **B** Mouse BMMNCs were infected with PD-1H shRNA (control) or pCDH-GFP-PD-1H-His_6_ overexpressing lentivirus followed by GFP flow cytometry sorting. Cell lysates were incubated with Ni-NTA agarose beads and the pull-down complex from control or PD-1H-His_6_ overexpressing BMMNCs was analyzed by LC/MS/MS Mass spectrometry (*n* = 3, with each sample analyzed twice). Figure was created with BioRender.com (**A**). Cluster analysis of the proteins that bind to PD-1H-His_6_ are shown (**B**). **C** Functional annotation charting of top 75 proteins enriched in PD-1H-His_6_ pull-down samples are listed. **D** BMMNCs from WT or *Pd-1h*^*-/-*^ mice were cultured in OCL differentiation medium without or with MMP-13. Activated Rac1 was detected in Rac1 pull-down complex from whole-cell lysates. Bands densitometry intensities from repeated experiments were shown. The experiment was performed three times with similar observations. **E** WT or *Pd-1h*^*-/-*^ OCLs differentiated without or with MMP-13 were stained with anti-Rac1 (green), anti-PD-1H (red), Acti-stain 670 phalloidin (gray) and DAPI (blue) and analyzed by confocal microscopy. Scale bar, 100 μm. Rac1/F-actin co-localization cell numbers per well were calculated (*n* = 3 wells per group). Mean ± SEM. *P ≤ 0.05 by one-way ANOVA.

While the T-cell inhibitory role of PD-1H is known^5,6,15^, the PD-1H binding proteins that control immune response are largely unknown. To identify interacting proteins, PD-1H-His_6_ recombinant protein was overexpressed in mouse bone marrow MNCs, bound protein complexes isolated from PD-1H-His_6_-transduced cell lysates by Ni-NTA agarose beads and the captured proteins identified by mass spectrometry (Fig. 4A). Since PD-1H has a high histidine content^16^, lysates were prepared from PD-1H knockdown cells to eliminate endogenous PD-1H from the pull-down control group. Cluster analysis of the bound proteins are shown in Fig. 4B. Functional annotation charting of the top 75 proteins enriched in PD-1H pull-down samples (with signal ratio of PD-1H-His_6_ pull-down *vs* control >2) indicate that almost 30% of the interacting targets were either cytoskeletal or cytoskeleton-associated proteins (Fig. 4C, Supplemental Data File 3).

While examining the mechanisms by which MMP-13/PD-1H signaling affects the reorganization of the F-actin cytoskeleton, we found that MMP-13 activates the Rho GTPase, Rac1, in WT, but not *Pd-1h*^*-/-*^, OCLs (Fig. 4D). Of note, *Pd-1h*^*-/-*^ OCLs also displayed decreased baseline Rac1 activation relative to WT controls. Immunofluorescence staining further indicated that PD-1H and Rac1 co-localize, especially at the F-actin belt in WT OCLs (Fig. 4E). Consistent with the decreased Rac1 activation and F-actin belt formation observed in *Pd-1h*^*-/-*^ OCLs, Rac1 failed to localize at F-actin-rich areas in *Pd-1h* knockout cells (Fig. 4E).

**Fig. 5:**
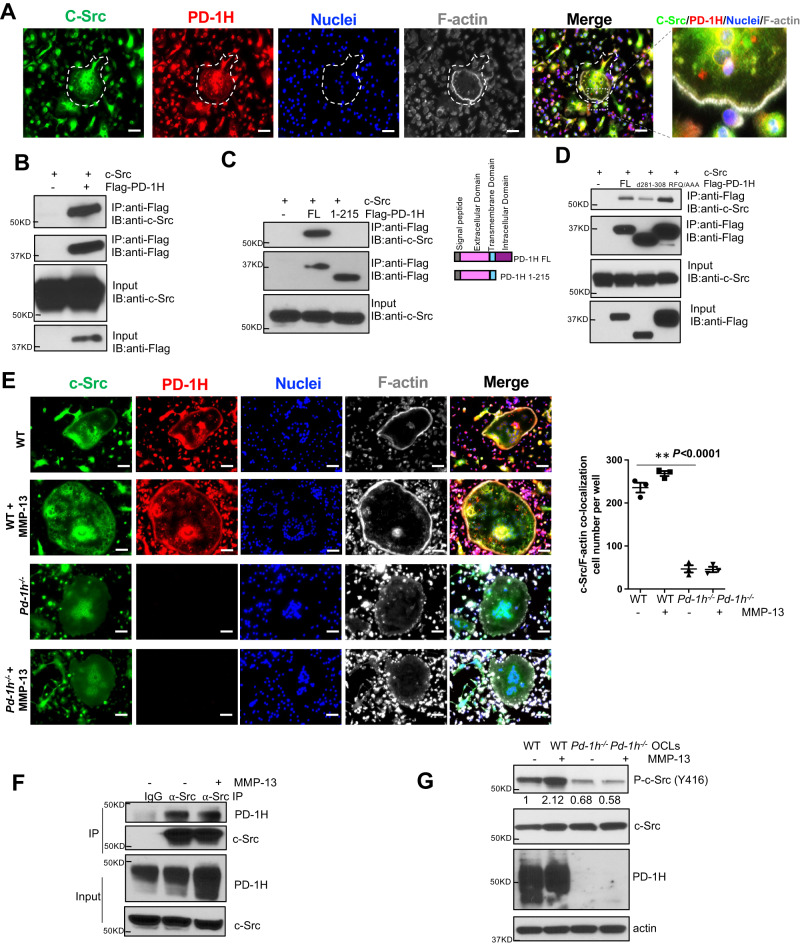
PD-1H binds to c-Src and mediates MMP-13 induced OCL activation. **A** WT OCLs differentiated without or with MMP-13 were stained by anti-c-Src (green), anti-PD-1H (red) and Acti-stain 670 phalloidin (gray) and DAPI (blue) and analyzed by confocal microscopy. Scale bar, 100 μm. The experiment was performed three times with similar observations. **B**–**D** c-Src was co-transfected in HEK-293 cells without or with Flag-PD-1H followed by co-IP by anti-Flag antibody (**B**), without or with Flag-PD-1H FL or extracellular domain (aa1-215) followed by co-IP by anti-Flag antibody (**C**), with Flag-PD-1H FL or △281-308 or R86A/F94A/Q95A triple point mutation (RFQ/AAA) followed by co-IP by anti-Flag antibody (**D**). The experiments were performed three times with similar observations. **E** WT or *Pd-1h*^*-/-*^ OCLs differentiated without or with MMP-13 were stained with anti-c-Src (green), anti-PD-1H (red), Acti-stain 670 phalloidin (gray) and DAPI (blue). Images were obtained by confocal microscopy. Scale bar, 100 μm. C-Src/F-actin co-localization cell numbers per well were calculated (*n* = 3 wells per group). **P ≤ 0.01 by one-way ANOVA. **F** RAW264.7 cells were differentiated into OCLs without or with MMP-13 followed by co-IP using IgG control or anti-PD-1H antibody. The experiment was performed three times with similar observations. **G** BMMNCs from WT or *Pd-1h*^*-/-*^ mice were cultured in OCL differentiation medium without or with MMP-13. Cell lysates were analyzed by western blotting with β-actin as loading control. Bands densitometry intensities from repeated experiments are shown. The experiment was performed three times with similar observations.

Given that the non-receptor tyrosine kinase, c-Src, associates with RANK and mediates RANKL-induced Rac1 activation and cytoskeleton reorganization^17,18^, we noted that c-Src and PD-1H almost completely co-localize on the F-actin sealing belt and perinuclear area (Fig. 5A). Co-IP assays confirmed the direct binding of c-Src to the C-terminal intracellular domain of PD-1H as a PD-1H^1–215^ mutant lacking its intracellular domain failed to bind c-Src (Fig. 5B, C). The c-Src binding site was further localized to the C-terminal end of PD-1H as deletion of aa 281-308 (△281-308) largely ablated its c-Src binding ability (Fig. 5D). In contrast, an R86A/F94A/Q95A triple point mutation (RFQ/AAA) in PD-1H, which blocked VISG3 binding to PD-1H^26^, failed to affect c-Src binding to PD-1H. IF staining of c-Src, PD-1H and F-actin indicated that in contrast to WT OCLs, c-Src in *Pd-1h*^*-/-*^ OCLs mainly accumulated in perinuclear areas, but not in the F-actin belt (Fig. 5E). Endogenous binding between c-Src and PD-1H was also confirmed in RAW 264.7 cells (Fig. 5F). Finally, whereas MMP-13 induced c-Src phosphorylation in WT OCLs, c-Src activation was impaired in *Pd-1h*^*-/-*^ cells (Fig. 5G).

We have shown previously that proteolytic activity is not required for MMP-13-induced OCL activation^3^. To confirm the dispensability of MMP-13 enzymatic activity in PD-1H-mediated OCL fusion, we treated both WT and *Pd-1h*^*-/-*^ OCLs with either the MMP-13 wild type zymogen or an enzymatically inactive MMP-13 E223A mutant. As shown in Supplemental Fig. 7, the MMP-13 E223A mutant induced an increase of osteoclast size and nuclei number to a degree similar to that observed with the WT MMP-13 zymogen in WT, but not *Pd-1h*^*-/-*^, OCLs.

### *Pd-1h* deficiency impairs MMP-13 induced-MM bone disease

**Fig. 6:**
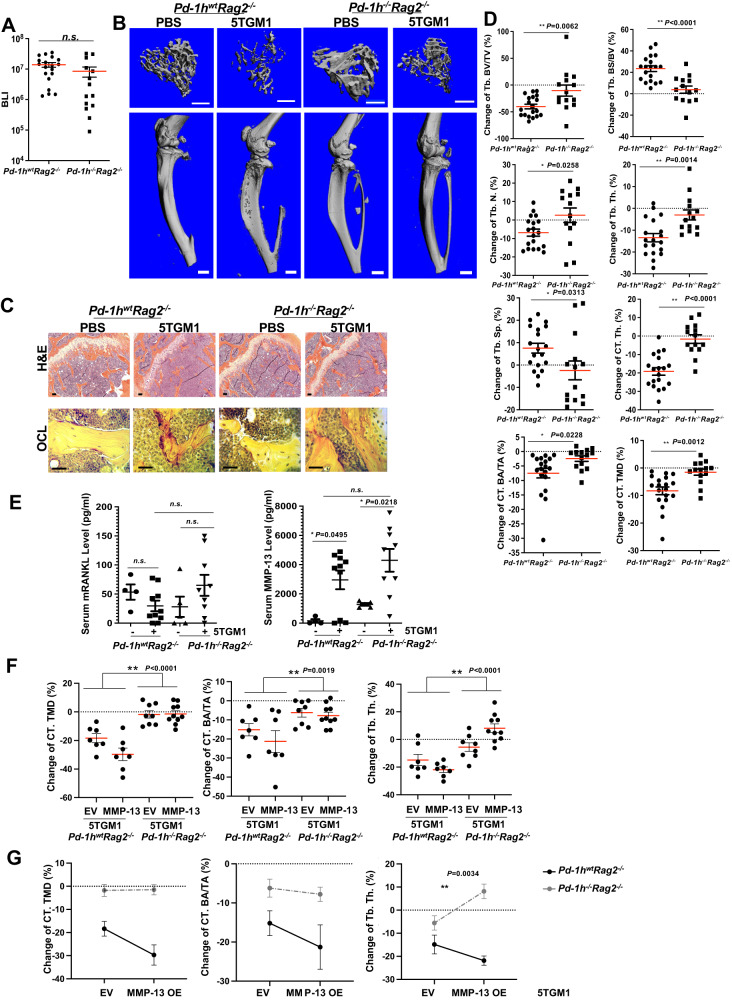
*Pd-1h*^*-/-*^ in recipient mice blocked 5TGM1 MM-induced bone lesion. **A** PBS or 2 × 10^5^ 5TGM1-luc cells were bilaterally intratibially injected into *Pd*-*1h*^*w*^^*t*^*Rag2*^*-/-*^ or *Pd-1h*^*-/-*^*Rag2*^*-/-*^ mice (8-12 wks, male). BLI total counts from tumor bearing tibiae were quantified for tumor growth. *Pd*-*1h*^*w*^^*t*^*Rag2*^*-/-*^ 5TGM1 bearing tibiae *n* = 19; *Pd-1h*^*-/-*^*Rag2*^*-/-*^ 5TGM1 bearing tibiae *n* = 14. Mean ± SEM by two-tailed student’s *t*-test. **B** Tibiae from **A** were analyzed by micro-qCT scanning. Representative 3D images of tibiae, adjacent femurs and tibiae trabecular bones from each treatment regimen are shown. Scale bar: upper panel 500 μm, lower panel 1 mm. **C** Tibiae from **B** were then decalcified and followed by H&E and TRAP staining. Scale bar, 100 μm. **D** Trabecular and cortical bone of mice tibiae from **B** were analyzed and relative changes in % compared to PBS injections are shown for microarchitectural parameters: trabecular bone volume fraction (Tb. BV/TV), trabecular bone surface to bone volume ratio (Tb. BS/BV), D.T. trabecular number (Tb. N.), D.T. trabecular thickness (Tb. Th.), D.T. trabecular spacing (Tb. Sp.), cortical bone thickness (CT. Th.), average cross-sectional area of cortical bone (CT. BA/TA), and cortical tissue mineral density (CT. TMD). *Pd*-*1h*^*w*^^*t*^*Rag2*^*-/-*^ 5TGM1 bearing tibiae *n* = 19; *Pd-1h*^*-/-*^*Rag2*^*-/-*^ 5TGM1 bearing tibiae *n* = 14. Mean ± SEM. *P ≤ 0.05, **P ≤ 0.01 by two-tailed student’s *t*-test. **E** Mice serum RANKL and MMP-13 levels were quantified by ELISA. *Pd*-*1h*^*w*^^*t*^*Rag2*^*-/-*^ control mice *n* = 4; *Pd*-*1h*^*w*^^*t*^*Rag2*^*-/-*^ 5TGM1 injected mice *n* = 10; *Pd-1h*^*-/-*^*Rag2*^*-/-*^ control mice *n* = 5; *Pd-1h*^*-/-*^*Rag2*^*-/-*^ 5TGM1 injected mice *n* = 9. Mean ± SEM. *P ≤ 0.05, **P ≤ 0.01 by one-way ANOVA test. **F** PBS or 2 × 10^5^ 5TGM1-luc EV or MMP-13 overexpression cells were intratibially injected into *Pd*-*1h*^*w*^^*t*^*Rag2*^-/-^ or *Pd-1h*^-/-^*Rag2*^-/-^ mice (13–16 wks, female). 3 weeks after tumor inoculation, tibiae were fixed for micro-qCT analysis as in **D**. *Pd*-*1h*^*w*^^*t*^*Rag2*^*-/-*^ 5TGM1 EV tumor bearing tibiae *n* = 7; *Pd*-*1h*^*w*^^*t*^*Rag2*^*-/-*^ 5TGM1 MMP-13 OE bearing tibiae *n* = 7; *Pd-1h*^*-/-*^*Rag2*^*-/-*^ 5TGM1 EV bearing tibiae *n* = 8; *Pd-1h*^*-/-*^*Rag2*^*-/-*^ 5TGM1 MMP-13 OE bearing tibiae *n* = 10. Mean ± SEM. *P ≤ 0.05, **P ≤ 0.01 by two-way ANOVA. **G** Same data from **F** are presented by interaction graphs. Mean ± SEM. *P ≤ 0.05 by two-way ANOVA.

To determine the role of MMP-13/PD-1H in the development of myeloma-induced lytic bone lesions, we developed an intratibial MM bone disease model using *Pd-1h*^*wt*^*Rag2*^*-/-*^ mice that recapitulates myeloma tumor growth coupled with severe osteolysis^3^. *Pd-1h*^*-/-*^*Rag2*^*-/-*^ double knockout mice were generated by crossing *Pd-1h*^*-/-*^ C57BL/6 with *Rag2*^*-/-*^ C57BL/6 mice. 2 × 10^5^ firefly luciferase-expressing 5TGM1 (5TGM1-luc) myeloma cells were bilaterally intratibially injected into age and sex paired *Pd-1h*^*wt*^*Rag2*^*-/-*^ or *Pd-1h*^*-/-*^*Rag2*^*-/-*^ mice. Three weeks following intratibial tumor injection, tumor growth was monitored by BLI (Fig. 6A), and tibiae were harvested for quantitative micro-CT, followed by histological analysis^3^. Data showed that 5TGM1-luc myeloma cells induced extensive lytic lesions and trabecular bone loss in *Pd-1h*^*wt*^*Rag2*^*-/-*^ but not in *Pd-1h*^*-/-*^*Rag2*^*-/-*^ mice with maintenance of overall bone structure (Fig. 6B, C). Quantitative analyses by micro-CT of trabecular and cortical bones confirmed that *Pd-1h*^*-/-*^*Rag2*^*-/-*^ recipient mice exhibited significantly less 5TGM1-induced bone loss relative to *Pd-1h*^*wt*^*Rag2*^*-/-*^ mice (Fig. 6D; Supplemental Fig. 8). As expected, while enlarged TRAP^+^ OCLs were observed adjacent to 5TGM1 tumors in *Pd-1h*^*wt*^*Rag2*^*-/-*^ mice, only small TRAP^+^ OCLs were detected in *Pd-1h*^*-/-*^*Rag2*^*-/-*^ mice (Fig. 6C). In contrast, osteoblast number did not differ between *Pd-1h*^*wt*^*Rag2*^*-/-*^ and *Pd-1h*^*-/-*^*Rag2*^*-/-*^ mice, suggesting that abrogated osteoclast activity protects *Pd-1h*^*-/-*^*Rag2*^*-/-*^ mice from myeloma-induced bone loss (Supplemental Fig. 9). Serum levels of both RANKL and MMP-13 were comparable between 5TGM1 bearing *Pd-1h*^*wt*^*Rag2*^*-/-*^ and *Pd-1h*^*-/-*^*Rag2*^*-/-*^ mice, which indicates that abrogated bone resorption in *Pd-1h*^*-/-*^*Rag2*^*-/-*^ mice did not occur as a consequence of reduced levels of osteoclastogenic factors (Fig. 6E). These data suggest that the failure of 5TGM1 myeloma cells to induce severe bone lesions in *Pd-1h*^*-/-*^ mice is mediated by PD-1H and confirms its critical role in OCL activation as well as the development of lytic bone lesions in MM.

To further investigate the in vivo role of PD-1H in MMP-13-driven MM bone disease, we injected either PBS, EV-transduced 5TGM1-GFP-Luc or MMP-13-overexpressing (OE) 5TGM1-GFP-Luc into *Pd-1h*^*wt*^*Rag2*^*-/-*^ and *Pd-1h*^*-/-*^*Rag2*^*-/-*^ mice and compared bone lesion progression by micro-qCT. As shown in Fig. 6F,G and Supplemental Fig. 10, compared to EV 5TGM1, overexpression of MMP-13 in 5TGM1 further enhanced the 5TGM1-induced bone disease in both trabecular and cortical bones as demonstrated by the decreased cortical bone tissue mineral density (CT. TMD) and average cross-sectional area of cortical bone (CT. BA/TA) as well as trabecular bone thickness (Tb. Th.). However, these effects were abrogated in *Pd-1h*^*-/-*^*Rag2*^*-/-*^ mice, further demonstrating the critical role of MMP-13 in *Pd-1h*-dependent bone destruction.

## Discussion

Bone disease is a devastating complication of multiple myeloma where it is characterized by osteoclast over-activation^2^. This hyperactivated state is mediated by MM cells producing osteogenic factors supported at sites adjacent to active bone resorption^2^. Our previous studies demonstrated that MMP-13 is a critical osteoclastogenic factor that is highly expressed by human MM cells^3^. MMP-13 is initially synthesized and secreted as an inactive zymogen until the pro-peptide domain is removed, thereby generating an active proteinase that participates in extracellular matrix degradation^19^. Distinct from its role in tumor progression and tissue remodeling, MMP-13 proteolytic activity is not required for a series of inductive effects, including promoting OCL multinucleation and bone resorption^3^. These findings led us to posit a new role for MMP-13 as a signaling molecule that interacts with a previously unidentified receptor on the OCL or OCL progenitor cell surface. Indeed, in a screening effort designed to detect the MMP-13 binding proteins in bone marrow mononuclear cells, we identified the co-inhibitory T-cell receptor homolog, PD-1H, as a cell surface-associated MMP-13 binding protein. Our findings identify the role of PD-1H in bone biology outside of its known immunoregulatory functions.

PD-1H is a type I transmembrane protein and member of the B7 family of negative checkpoint regulators^5,6,15^. PD-1H potentially acts either as a ligand or a receptor on T-cells to inhibit T-cell effector function and maintain peripheral tolerance^6,15^. Nevertheless, little is known with regard to its corresponding receptors or ligands^5,6,15,20–22^. Recently, Johnston et al. reported that PD-1H can act as a ligand that engages and suppresses T cells selectively at acidic pH by binding to its receptor, PSGL-1^23^. Others identified PD-1H as a potential receptor for VSIG-3 that similarly serves to regulate immune responses by inhibiting T-cell proliferation. However, the latter study was based solely on in vitro assays using a VSIG3 extracellular domain-Fc recombinant protein^24^, and data from other groups failed to detect VSIG3/PD-1H binding in cell-based assays^23,25^. Even so, whereas a three amino acid epitope (R86/F94/Q95) in the PD-1H ECD has previously been reported to be responsible for VISG3 binding^26^, the MMP-13 binding site on PD-1H is distinct from this epitope as the triple point mutation did not affect MMP-13 binding (see Supplemental Fig. 11). A more recent study suggested that VSIG3 regulates OCL differentiation, however, this occurred via a homophilic interaction that operates independently of PD-1H^25^, thereby excluding heterotypic interactions between VSIG3 and PD-1H. Consistently, a recombinant PD-1H extracellular domain (ECD) failed to affect OCL differentiation^25^.

OCLs undergo a unique and highly dynamic F-actin rearrangement during differentiation and bone resorption. At early stages, OCLs form integrin-based, F-actin-rich podosomes that eventually evolve into sealing zones that support bone resorption by confining OCL-secreted protons and proteases to the surface of the bone matrix^9,10,27,28^. By mass spectrometry, we found that PD-1H-binding proteins in OCLs are enriched for cytoskeleton-associated proteins that control F-actin assembly, cell adhesion, polarization, and migration (Supplemental Fig. 12). Whereas PD-1H localized to filopodial spikes in WT OCLs where it co-localized with F-actin, in *Pd-1h*^*-/-*^ OCLs, podosome assembly was significantly diminished, resulting in decreased numbers of podosome clusters and F-actin ring/belt-forming cells. Interestingly, 3-D imaging indicated that *Pd-1h*^*-/-*^ OCLs tend to display decreased Z-axis height, less cell polarity, and irregular shapes/spikes. Consistently, bone resorption activity of *Pd-1h*^*-/-*^ OCLs was significantly impaired. But most important is our finding that MMP-13 only enhances F-actin ring/belt formation and bone resorption in the presence of PD-1H in WT, but not *Pd-1h*^*-/-*^, OCLs. As such, this study identified a critical role for PD-1H in OCL formation and MMP-13-mediated bone resorption in multiple myeloma. In line with our findings of a potential role of PD-1H in MM bone disease is the finding that the gene expression level of PD-1H (MMRF CoMMpass study IA17 dataset) (https://research.themmrf.org) is significantly higher in MM patients with bone pain, reflecting active MM bone disease (mean: 139.2 TPM, *N* = 201) compared to those without bone pain (mean: 120.7 TPM, *N* = 111) (*P* = 0.0277 by two-tailed student’s t-test), supporting the proposition that PD-1H levels positively correlate with active bone disease in MM.

**Fig. 7:**
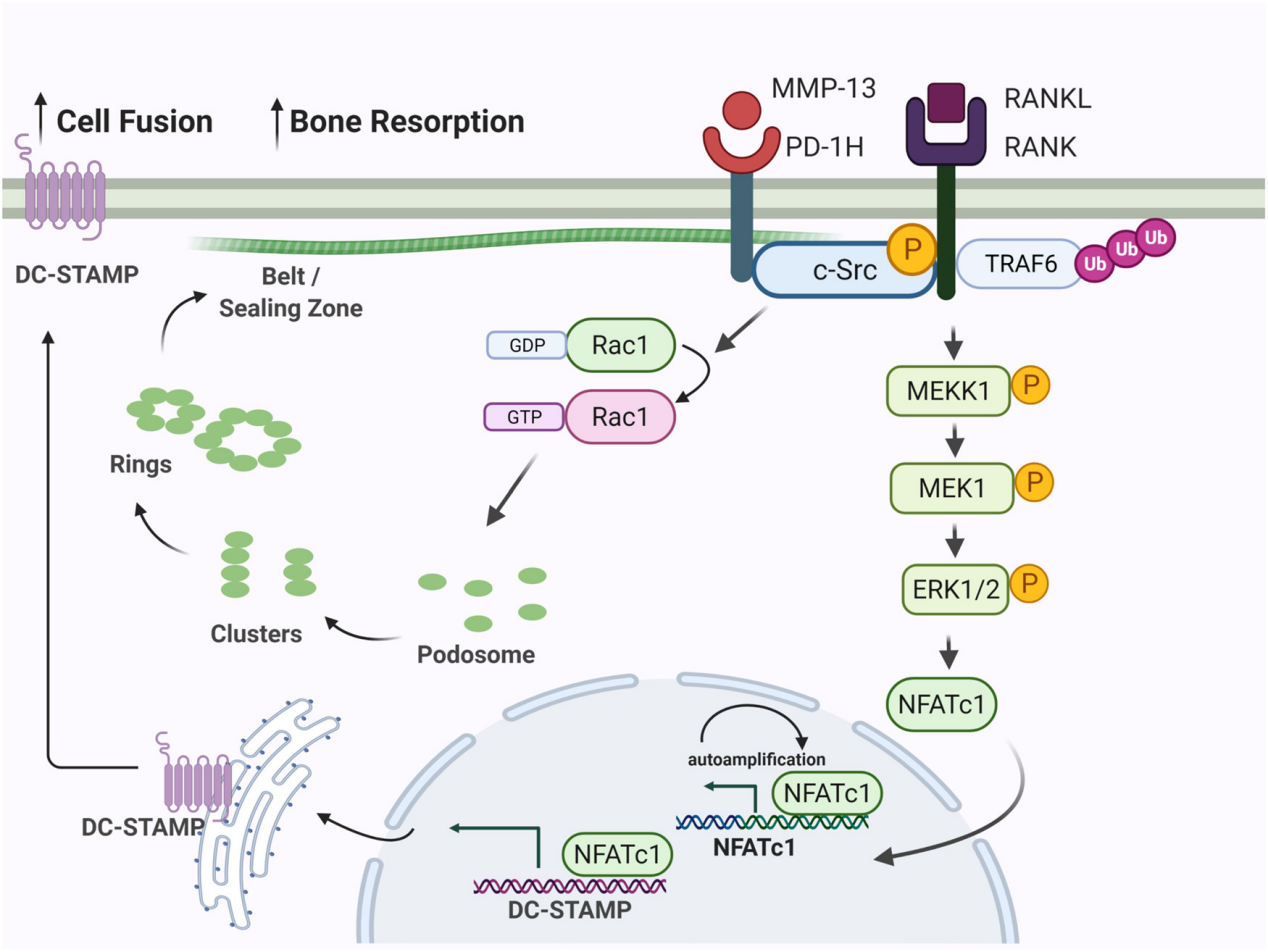
MMP-13 binds PD-1H expressed on OCL precursors and induces osteoclast formation and activation. MMP-13 secreted by MM cells binds to PD-1H on OCL cell surface. The binding enhances RANKL triggered activation of c-Src and subsequently ERK1/2 mediated upregulation of NFATc1 and DC-STAMP, resulting in increased cell fusion. In addition, PD-1H directly associates with cytoskeleton protein c-Src and activates Rac1, hence regulates cytoskeleton reorganization. Figure was created with BioRender.com.

Despite an extensive body of literature linking PD-1H function to immune regulation, a role in the bone homeostasis such as bone resorption, has not been described and moreover, the binding partners of PD-1H are largely unknown. Here, we demonstrate a direct association of c-Src with PD-1H. c-Src is known to serve as a central hub in OCL signaling networks by activating downstream kinase pathways, including MAPK and PI3K/AKT, while upregulating NFATc1 and DC-STAMP to promote OCL differentiation and fusion. In addition, c-Src plays a critical role in cytoskeleton reorganization where it localizes to the sealing zone in mature OCLs, and regulates podosome dynamics, in part, by activating Rac1^29^. Supporting its unique function in OCL biology, we found that c-Src expression was dramatically elevated during OCL differentiation. As shown in Supplemental Fig. 13, c-Src level was not detectable in BMMNCs and pre-OCL on day 1 of differentiation but was elevated at day 2 and peaked at day 4 when OCLs mature, explaining why c-Src was not detected in the PD-1H pull-down complex from the day 1 pre-OCL lysates (Supplemental Data File 3). As expected, co-IP assays and immunofluorescence staining of mature OCLs at days 3 and 4 demonstrated the direct interaction and co-localization of PD-1H and c-Src in mature OCLs. Our data suggest, therefore, that MMP-13 promotes c-Src signaling via a PD-1H-dependent process (Fig. 7).

Given our newly identified role for the MMP-13/PD-1H signaling axis in OCLs, we sought to define the importance of PD-1H in a mouse MM model where we have previously identified MMP-13 as a key player in triggering OCL-mediated bone resorption in vivo. Compared to *Pd-1h*^*wt*^*Rag2*^*-/-*^ mice, *Pd-1h*^*-/-*^*Rag2*^*-/-*^ mice are significantly less vulnerable to 5TGM1 MM-induced bone loss, confirming the role of PD-1H in MM bone disease in vivo. Interestingly, *Pd-1h*^*-/-*^*Rag2*^*-/-*^ male mice demonstrated developmental deficiencies with significantly lower body weight, size and bone volume compared to *Pd-1h*^*wt*^*Rag2*^*-/-*^ male mice (Supplemental Fig. 8). This effect seems to be sex-dependent since these changes were less profound in the female mice cohort (Supplemental Fig. 10). Lineage-specific deletion of *Pd-1h* in a conditional knockout mouse model is the focus of future research to further delineate the role of the *Pd-1h* and MMP-13/PD-1H axis in osteoblast and osteoclast biology as well as skeletal development.

Beyond OCLs or their progenitors, PD-1H is expressed on multiple cells of hematopoietic origin, including T cells, neutrophils, monocytes, macrophages, and dendritic cells^5,6,30,31^. Given the critical role of PD-1H in T-cell inhibition and tumor immunosuppression, studies of its crosstalk with different cell types in the MM microenvironment are likewise deserving attention. Recent studies have identified roles for OCLs in skewing immunologic responses towards either immune suppression or inflammation depending on the conditions studied^32–36^. Further, OCLs share many similarities with macrophages and dendritic cells, and likewise display phagocytic properties^35^ as well as the ability to process, present and cross-present antigens resulting in T-cell activation^33,34^. In MM patients, OCLs express checkpoint molecules such as PD-L1 and IDO that may contribute to T-cell inhibition and immune supression^32,36^. From this perspective, our findings may lead to novel therapeutic strategies to treat both MM bone disease and overcome myeloma-induced immunosuppression by targeting the MMP-13/PD-1H axis.

## Methods

### Ethics statement

The reported research complies with all relevant ethical regulations. All animal procedures were reviewed and approved by the IACUC of Columbia University, New York (Protocols AC-AAAE9803 and AC-AAAW6454). The human cord blood samples were purchased from a commercial vendor Upstate State University of New York Upstate Medical Center’s Upstate Cord Blood Bank (Syracuse, NY) as de-identified discarded human material. The purchase was made under an MTA agreement between Columbia University and the Research Foundation for the SUNY (Syracuse, NY).

### Mice

C57BL/6 WT and *Rag2*^*-/-*^ mice were purchased from Jackson Laboratory (Bar Harbor, Maine). C57BL/6 *Pd-1h*^*-/-*^ mice were obtained from Mutant Mouse Resource & Research Centers (MMRRC) (Davis, CA)^15^. *Pd-1h*^*-/-*^*Rag2*^*-/-*^ mice were generated by crossbreeding C57BL/6 *Pd-1h*^*-/-*^ and C57BL/6 *Rag2*^*-/-*^ mice. Mice were housed with food (PicoLab Rodent Diet 20, Cat# 5053) and water ad libitum in a temperature and humidity-controlled environment on a 12-h light–dark cycle. All experimental animals were used at 8-16 weeks of age as indicated in the individual experiments. Sex of the experimental mice are indicated in the individual experiment.

### Cell lines

The murine MM cell line 5TGM1-GFP was kindly provided by Dr. G. David D. Roodman from Indiana University, and then transduced with MSCV-Luciferase-RFP-TK lentivirus (Cat#BLIV302, System Biosciences) to generate the 5TGM1-luciferase-expressing cells. RAW264.7 cell line was purchased from ATCC (Cat#TIB-71). Both cell lines were cultured in RPMI1640 medium with L-glutamine, penicillin-streptomycin, and 10% FBS. HEK 293T-cell line was purchased from Takara and cultured in DMEM medium with L-glutamine, penicillin-streptomycin, and 10% FBS.

### Human CD34 cells

De-identified cord blood samples were purchased from the State University of New York Upstate Medical Center’s Upstate Cord Blood Bank Program. Mononuclear cells were isolated using Ficoll-Paque Plus (Cytiva). CD34 cells were then purified using human CD34 MicroBead kit (Miltenyi Biotec) following the manufacturer’s protocol and cultured in CD34 expansion medium (StemSpan SFEMII (Stemcell Technology), 50 ng/ml SCF, IL-3, IL-6, TPO, FLT3L (Peprotech) and 500 nM UM729 (Stemcell Technology)).

### Pull-down and mass spectrum assay

Non-adherent bone marrow mononuclear cells isolated from WT C57BL/6 mice were cultured with M-CSF (100 ng/ml) for 1 day, and cell lysates collected and incubated with pro-MMP-13-Myc-His_6_ recombinant protein at 4 °C for 4 h, followed by incubation with Ni-NTA beads (Qiagen, Germany) for affinity pull-down. MMP-13 binding proteins were extracted after extensive washing by PBS buffer and analyzed by mass spectrometry. Briefly, the proteins recovered from pull-downs were precipitated with chloroform and methanol, solubilized using 0.1% Rapigest (Waters Corp., Milford MA), and digested with trypsin (6 ng/µl) at 37 °C overnight. Peptides were separated with a 0.75 µm ID x 25 cm reverse phase 1.7 µm particle diameter C18 (HSS T3) column (Waters Corp.) on a NanoAcquity liquid chromatograph coupled to a Synapt G2 HDMS mass spectrometer (Waters Corp,) as described previously^37^. Two 120 min liquid chromatography (LC) /mass spectrometry (MS) runs were performed for each biological replicate with data collection in resolution/ion mobility mode. Spectra were analyzed with ProteinLynx Global Server (Vers.2.5, RC9) (Waters Corp.). Accurate mass and retention time matches of precursors were made across all LC/MS runs for label-free intensity-based quantitation were performed with Rosetta Elucidator software V. 4.0.0.2.31) (Ceiba Solutions, Inc.) as described previously^37^ with protein database searches against Mouse UniProt reviewed database with isoforms on Mascot server V. 2.5.1.

For PD-1H pull-down assay, non-adherent bone marrow cells were transduced with a pCDH-GFP-PD-1H-His_6_ (System Biosciences) lentivirus that expresses a PD-1H-His_6_ recombinant protein. Positively transduced cells were sorted by GFP-based flow cytometry and cell lysates were collected followed by Ni-NTA beads affinity pull-down. PD-1H-His_6_-binding proteins were extracted and analyzed as described above. PD-1H shRNA lentivirus-infected cell lysates were pulled down by Ni-NTA beads as control. Each treatment was performed in triplicate, and each replicate was analyzed in duplicate. Separations of peptides from protein digests were performed with an Ultimate 3000 RSLCnano liquid chromatograph and a 75 μm ID x 50 cm Acclaim PepMap C18 column with an acetonitrile/formic acid gradient as described previously^38^. Mass spectra were collected with a Q Exactive HF mass spectrometer (Thermo Scientific) in positive ion mode using data-dependent acquisition. Data analysis was performed with Mascot and Elucidator software for label-free quantitation^38^.

### Osteoclast formation and bone resorption assays

Non-adherent bone marrow mononuclear cells isolated from WT or *Pd-1h*^*-/-*^ C57BL/6 mice were cultured in α-MEM supplemented with 10% FBS, 50 ng/ml mM-CSF and 50 ng/ml mRANKL (R&D Systems, Minneapolis, MN) in the presence or absence of recombinant human pro-MMP-13 for 4 d followed by TRAP staining^3^. For bone resorption pit assays, bone marrow mononuclear cells were seeded on bovine femur slices (Immunodiagnostic Systems, United Kingdom) treated as described above. Osteoclast formation on dentin slices was confirmed by TRAP staining and bone resorption lacunae identified following hematoxylin staining^3^. Bone resorption area was quantified using Image J^3^.

### Immunofluorescence staining and confocal microscopy

Non-adherent mononuclear bone marrow cells were seeded atop tissue culture-treated glass slides (Corning, NY) and cultured in vitro to monitor OCL differentiation. On day 4, cells were fixed by 4% PFA and stained with Acti-Stain 488 Phalloidin (for F-actin) (100 nM, Cat#PHDG1, Cytoskeleton, Denver, CO), PE-anti-PD-1H (1: 200 dilution, Cat#150204, Biolegend, San Diego, CA), and Alexa Fluor647-anti-cortactin (1:100 dilution, Cat#ab202650, Abcam, Cambridge, MA), Alexa Fluor647-anti-vinculin (1:100 dilution, Cat#ab196579, Abcam), or Acti-Stain 670 Phalloidin (for F-actin) (100 nM, Cat#PHDN1, Cytoskeleton). Nuclei were stained by DAPI. For c-Src and Rac1 staining, rabbit-anti-c-Src (1:100 dilution, Cat#2109, Cell Signaling Technology, Danvers, MA) or mouse-anti-Rac1 (1:100 dilution, Cat#16118, Thermo Fisher Scientific, Waltham, MA) and Alexa Fluor 488-conjugated goat anti-rabbit or anti-mouse secondary antibodies (1:300 dilution Cat#A-11034; Cat#A-21202, Invitrogen, Carlsbad, CA) were used. Images were obtained by Leica Fluorescent Microscope or Nikon Ti Eclipse inverted confocal microscopy. X-Z and Y-Z planes of F-actin clusters and actin rings were analyzed by NIS Elements Software (NIKON). Co-localization of PD-1H with F-actin on sealing zones was visualized by 3-D stacking with NIS Elements Software.

### Co-IP and immunoblotting

Whole cell extracts of transfected HEK293 cells were prepared by lysing cells in RIPA buffer (Sigma-Aldrich) supplemented with protease inhibitor cocktail (Cell Signaling Technology). The cells extracts were used for immunoprecipitation (IP), and immunoblotting (WB) was performed following the standard protocol^3,7,39,40^ using antibodies purchased from the following vendors: anti-MMP-13 (1:6000 dilution, Cat#39012, Abcam); anti-m-PD-1H and anti-h-PD-1H (1:2000 dilution, Cat# AF7005 and AF7126, R&D Systems); anti-h/m-PD-1H (for IP and WB) (1:50 dilution for IP; 1:2000 dilution for WB, Cat#54979, Cell Signaling Technology); anti-Flag tag (1:2000 dilution, Cat# 3165, Sigma-Aldrich); anti-c-myc tag (1:2000 dilution, Cat#sc-40, Santa Cruz Biotechnology); anti-DC-STAMP (clone 1A2) (1:2000 dilution, Cat#MABF39, Millipore); anti-NFATc1 (7A6) (1:2000 dilution, Cat#sc-7294, Santa Cruz biotechnology); anti-P-ERK1/2(D13.14.4E), and anti-ERK1/2, anti-P-c-Src (Y416), and anti-c-Src: (1:2000 dilution, Cat#4370; 9102; 2101; 2123; Cell Signaling Technology); and β-actin (1:6000 dilution, Cat#A5441, Sigma-Aldrich). Rac1 activation was determined using Active Rac1 Pull-Down and Detection Kit (Cat#16118, Thermo Fisher Scientific).

### Real-time PCR analysis

Total RNA was isolated with TRIZOL reagent and cDNA generated with SuperScript III reverse transcriptase (Cat#18080400; Invitrogen, Grand Island, NY), followed by Taqman (Invitrogen) based real-time polymerase chain reaction assays (Cat# 4352042; Invitrogen, Grand Island, NY) performed as described^3,7,41^. TaqMan® Gene Expression Assay (Cat# 4331182) was used for m-Pd-1h: Mm00472314_m1; m Dc-stamp: Mm04209236_m1; m-beta-actin: Mm00607939_s1.

### Expression constructs

MMP-13 WT (FL), E223A, Cat and Hem constructs were reported previoulsy^3^. WT mouse PD-1H cDNA was generated by PCR using cDNA prepared from WT C57BL/6 mouse BMCs as template using the following primers: forward: 5′-ACTGAGTTCGAACCATGGGTGTCCCCGCGGTCCCAGAG-3′ and reverse: 5′-ACTG CTCGAGTTAGATGGCTTCAGAGTTAGGGGA-3′. PCR products were then cloned into N13-Flag (GeneCopoeia) or pCDH-GFP lentiviral vector (System Biosciences). 6XHis tag was introduced into PCDH-GFP-Flag-m-PD-1H C-terminal by Q5 mutagenesis PCR using the primer pair: forward 5′-CACCACCACTAACTCGAGTGCGGCCGC-3′ and reverse 5′-ATGATGATGGATGGCTTCAGAGTTAGGGGAG-3′. Mouse PD-1H extracellular domain construct (ECD) was generated PCR using the primer pair: forward 5′-ACTGAGTTCGAACCATGGGTGTCCCCGCGGTCCCAGAG-3′ and reverse 5′-ACTGCTCGAGTTAAGCCGTGATGCTGTCACT-3′ with N13-flag-PD-1H as template and cloned into N13-Flag by similar strategy. Human PD-1H cDNA was generated by PCR using cDNA prepared from RPMI8226 cells with the following primers: forward 5′-ACTGAGTTCGAACCATGGGCGTCCCCACGGCCCTGGAG-3′ and reverse 5′-ACTGCGGCCGCCTAGATGACCTCAAAGTTTGGAGA-3′ and then cloned into N13-flag. Human PD-1H domain-deleted construct (aa1-215) was generated by primer pair forward 5′-ACTGAGTTCGAACCATGGGCGTCCCCACGGCCCTGGAG-3′ and reverse 5′-ACTGCGGCCGCCTAGACCAGGAGCAGGATGAG-3′ and cloned into N13-flag as the full-length PD-1H. PD-1H domain-deleted construct (d281-308) was generated by Q5 mutagenesis PCR using primer pair forward 5′-TAGGCGGCCGCAACCCAG-3′ and reverse 5′-CGAAAGCAGATGCCGCCC-3′. Human PD-1H R86A/F94A/Q95A point mutation was generated by 2 steps of Q5 mutagenesis PCR using the following primer pairs: R86A forward 5′-CTGCTCAGAGgcgcgcCCCATCCGCAACCTCAC-3′ and reverse 5′-GTCTGCACCTCGCCCCTC-3′; FQ94/95AA forward 5′-agatCTTCACCTGCACCATGGAG-3′ and reverse 5′-gcggcCGTGAGGTTGCGGATGGG-3′. Constructs were verified by sequencing with protein expression confirmed by Western blotting following transfection in HEK293 cells. Full-length c-Src in pCDNA3 construct was obtained from Addgene (Cat#42202, Addgene, Watertown, MA).

### Lentivirus infection and PD-1H knockdown

To silence PD-1H expression in mouse bone marrow cells, freshly isolated BMCs were cultured in MEM-alpha medium supplemented with 10% FBS, 1% P/S, 10 ng/ml IL-3, 10 ng/ml IL-6 and 10 ng/ml SCF overnight. On the second day, the medium was changed to MEM-alpha with 10%FBS, 1%P/S, 10 ng/ml IL-3, 50 ng/ml IL-6, 100 ng/ml SCF and 4 μg/ml polybrene, and incubated with pGreenpuro (System Biosciences) empty vector lentiviral control or PD-1H-targeting shRNA (5’-GGACGGTACCTGCTCTCTGAC-3’) lentiviral particles. Cells were selected by GFP sorting^42^. On day 14, cells were used for OCL differentiation assay. PD-1H protein and mRNA levels were determined in control and silenced cells by Western blotting and qRT-PCR, respectively.

To silence PD-1H in human OCL, CD34^+^ cells were infected by lentivirus pGreenpuro empty vector (EV) or sh-h-PD-1H (5′-GTCCCTGACTCTCCAAACTTTG-3′)^41^. 3 days later, GFP^+^ cells were selected by flow cytometry followed by expansion for 5 days. Cell medium were then changed into human OCL differentiation medium (α-MEM supplemented with 10% FBS, 50 ng/ml human M-CSF and 50 ng/ml human RANKL (R&D Systems, Minneapolis, MN)) and cultured for 21 days followed by WB and TRAP staining respectively^3^.

### Transwell assays

For MM-OCL Transwell co-culture assays, WT or *Pd-1h*^*-/-*^ mouse non-adherent bone marrow cells were cultured in the lower chambers of 24-well transwell plates in the OCL differentiation media. 5TGM1 EV or 5TGM1-MMP-13 KD cells were seeded in the upper chamber and co-cultured for 4 d^3^ followed by TRAP staining.

### 5TGM1 mice model

2 × 10^5^ 5TGM1-TK-GFP-luciferase (5TGM1-luc) EV or MMP-13 overexpressing murine myeloma cells were bilaterally injected into *Pd-1h*^*wt*^*Rag2*^*-/-*^ or *Pd-1h*^*-/-*^*Rag2*^*-/-*^ male mice tibiae. In vivo tumor growth was monitored by weekly BLI imaging using IVIS Spectrum Imaging System (PerkinElmer, MA). Mice tibiae were dissected 3 weeks after tumor injection and fixed in 10% neutral-buffered formalin buffer for 2 days.

### Micro-quantitative computed tomography

Trabecular bone from the proximal tibia and cortical bone at the tibial mid-diaphysis were scanned using a micro-qCT system (VivaCT40; Scanco Medical, Wangen-Brüttisellen, Switzerland). For trabecular microarchitecture, 100 slices, corresponding to a 1.05 mm region underneath the growth plate, were obtained with 10.5 μm spatial resolution. For the cortical bone, 50 slices, corresponding to a 1.05 mm region starting in a region that was 56% of the tibia length from its proximal end, were obtained with 21 μm spatial resolution. A global thresholding technique was applied to segment the grayscale image into binarized images. Both the trabecular and cortical compartments were analyzed with a semiautomatic contouring technique to assess microstructural parameters^3^. Three-dimensional reconstructions were generated from stacked two-dimensional images.

### Bone histology and immunohistochemical staining

Following micro-qCT, the same tibiae were decalcified in 10% EDTA buffer (pH 7.4) for 2 weeks at 4°C and embedded in paraffin. Bone sections (5 μm thickness) were stained with H&E and for tartrate-resistant acid phosphatase (TRAP) using a Leukocyte Acid Phosphatase Kit (Sigma-Aldrich). For OB staining, paraffin sections underwent 10 mM sodium citrate antigen retrieval, followed by staining with anti-osteocalcin Ab (1:100 dilution, ab93876; Abcam) and EnVision+ Systems HRP (DAB) (Dako, Santa Clara, CA) according to the manufacturer’s instructions^3^.

### Statistics

Quantitative data are presented as the means ± SEM or SD as indicated in individual experiments. Statistical significance was assessed by two-tailed Student’s *t*-test for comparisons between two groups. In experiments with more than two experimental groups, Bonferroni multiple comparison test for multiple comparisons was applied to pairwise comparisons subsequent to one-way or two-way ANOVA as indicated in individual experiments. *P* ≤ 0.05 was considered significant, and *P* ≤ 0.01 was considered highly significant. Graphs were generated using Graphpad Prism 9.

### Reporting summary

Further information on research design is available in the Nature Portfolio Reporting Summary linked to this article.

## Supplementary information

Supplementary Information

Description of Additional Supplementary File

Supplementary Dataset 1

Supplementary Dataset 2

Supplementary Dataset 3

Reporting Summary

## Data Availability

All raw mass spectrometry files produced in this work are publicly available at the MassIVE proteomics repository under accession MSV000089119: (10.25345/C5251FP6G). The PD-1H RNA-Seq data from MMRF CoMMpass study IA17 dataset are publicly available (https://research.themmrf.org and www.themmrf.org). All data generated or analyzed during this study are included within the article and its Supplementary Information files. Source data are provided with this paper.

## Source data

Source data
