# Supplementary material for: The checkpoint inhibitor PD-1H/VISTA controls osteoclast-mediated multiple myeloma bone disease
Source: Nat Commun. 2023 Jul 17;14:4271. doi: 10.1038/s41467-023-39769-8 (PMC10352288; doi:10.1038/s41467-023-39769-8)

Supplemental Figure 1

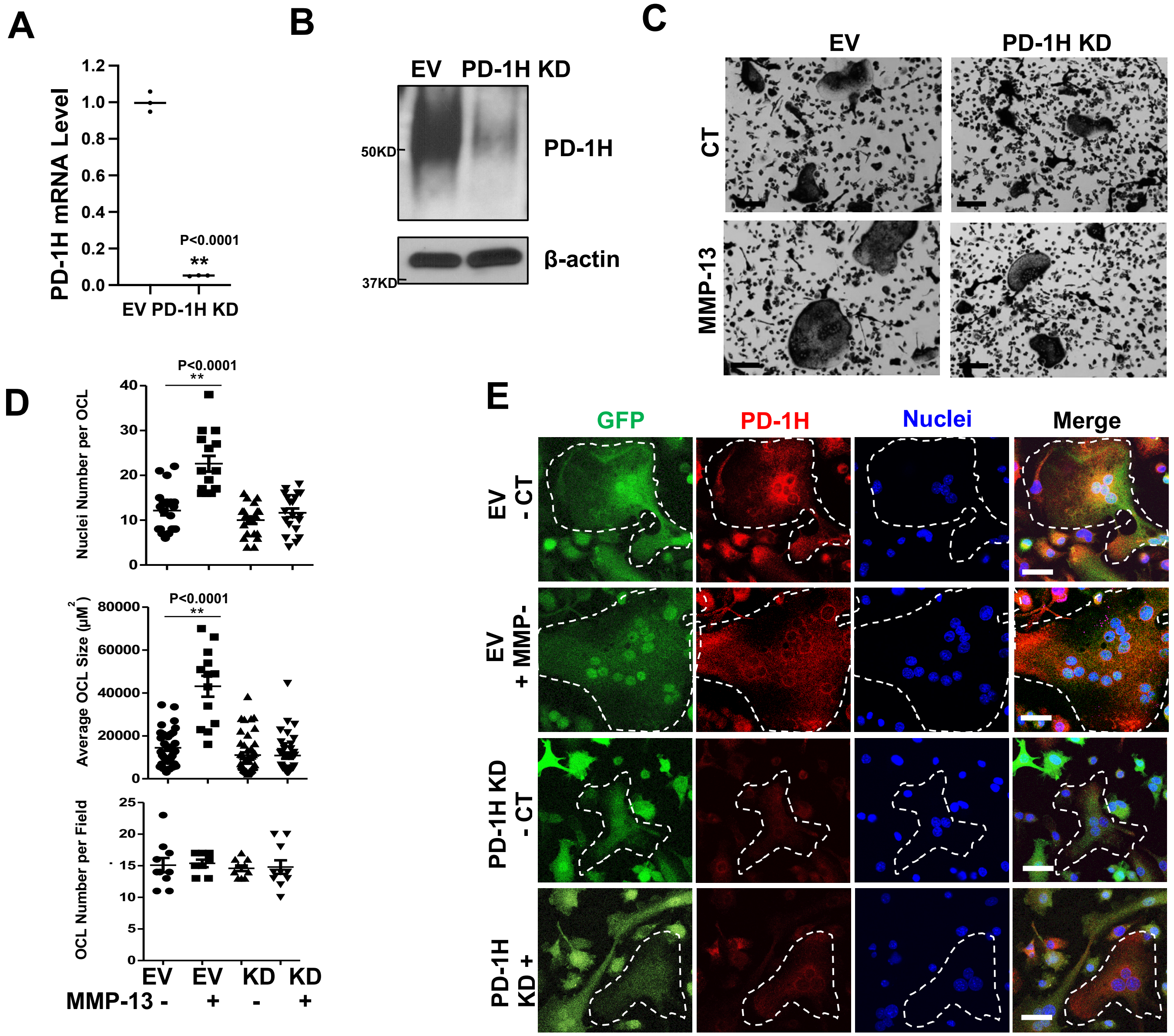

Supplemental Figure 1. PD-1H silencing blocks MMP-13 induced OCL fusion. (A-B) WT mice bone marrow cells (BMCs) were infected with pGreenpuro empty vector (EV) or m-PD-1H shRNA (KD) followed by GFP$^+$ flow cytometry sorting. PD-1H knockdown was confirmed by quantitative real time PCR (A) and western blotting (B). β-actin was used as internal control. For PCR, relative PD-1H mRNA levels were plotted as mean ± SD from n = 3 experimental replicates. $^{**}P \leq 0.01$ by two-tailed Student's *t* test. **(C-D)** BMCs from (A) were cultured in OCL differentiation medium without or with MMP-13 followed by TRAP staining. Scale bar, 100 μm (C). Nuclei number per OCL, average OCL size and OCL number per field were assessed by microscopy and ImageJ software. Nuclei number per OCL was calculated based on the randomly imaged fields of OCLs: EV control *n*=20 cells; EV+MMP-13 *n*=15 cells; KD CT *n*=20 cells; KD+MMP-13 *n*=20 cells. Average OCL size was measured based on the randomly imaged fields of OCLs: EV control *n*=35 cells; EV+MMP-13 *n*=14 cells; KD CT *n*=45 cells; KD+MMP-13 *n*=38 cells. OCL number per field was assessed by *n*=10 random fields. Mean ± SEM. $^{**}P \leq 0.01$ by one-way ANOVA (D). **(E)** BMCs from (A) were cultured in osteoclast differentiation medium without or with MMP-13 followed by immunostaining by FITC-anti-GFP and PE-anti-PD-1H. Nuclei were stained by DAPI. Scale bar, 50 μm. The experiment was repeated three times with similar results.

Supplemental Figure 2

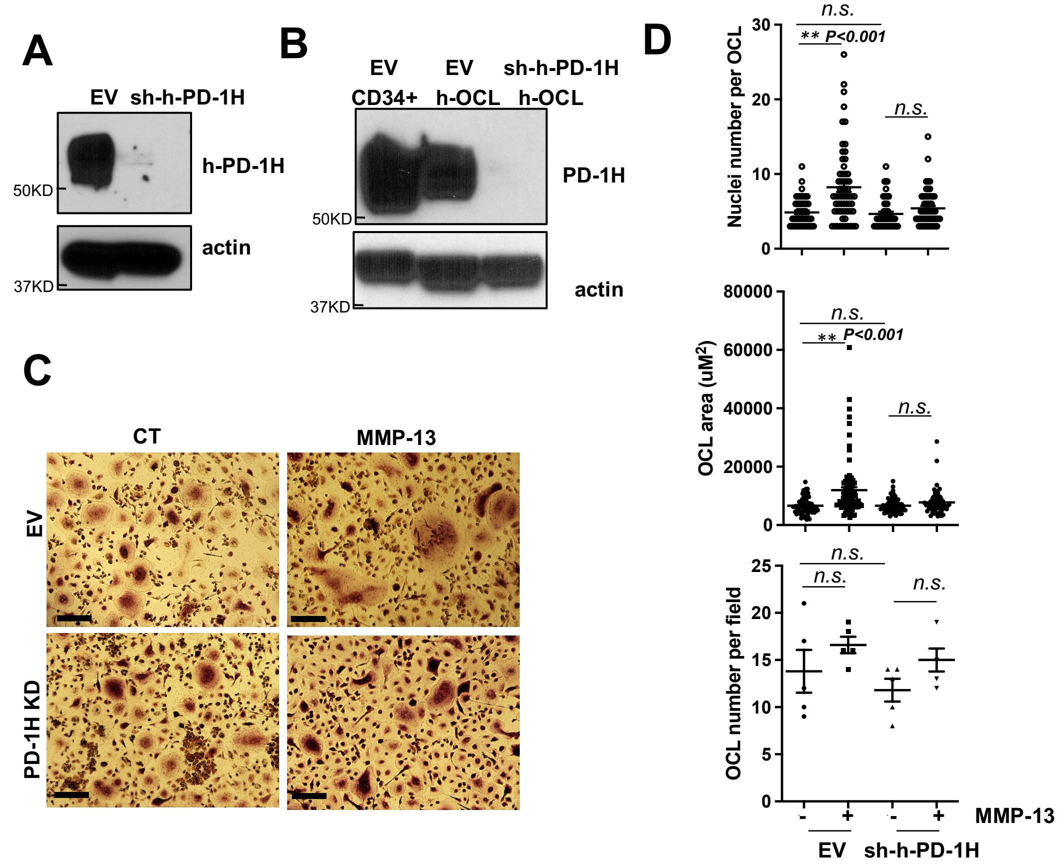

Supplemental Figure 2. PD-1H knockdown in human OCL abrogates MMP-13 induced OCL fusion. Human cord blood $CD34^+$ cells were infected with pGreenpuro empty vector (EV) or sh-h-PD-1H lentivirus. **(A)** GFP^+ from EV or sh-h-PD-1H infected $CD34^+$ cells were sorted followed by western blotting for h-PD-1H and actin. The experiment was repeated three times with similar results. **(B-D)** GFP^+ EV or sh-h-PD-1H infected $CD34^+$ cells were cultured for osteoclast differentiation followed by western blotting (B) or TRAP staining (C). Nuclei number per OCL, average OCL size and OCL number per field were assessed by microscopy and ImageJ software. Nuclei number per OCL was measured based on the randomly imaged fields of OCLs. EV control *n*=57 cells; EV+MMP-13 *n*=62 cells; KD CT *n*=49 cells; KD+MMP-13 *n*=55 cells. Average OCL size was measured based on the randomly imaged fields of OCLs: EV control *n*=68 cells; EV+MMP-13 *n*=70 cells; KD CT *n*=61 cells; KD+MMP-13 *n*=53 cells. OCL number per field was assessed by *n*=5 randomly imaged fields. Mean ± SEM. $**P \leq 0.01$ by one-way ANOVA (D).

Supplemental Figure 3

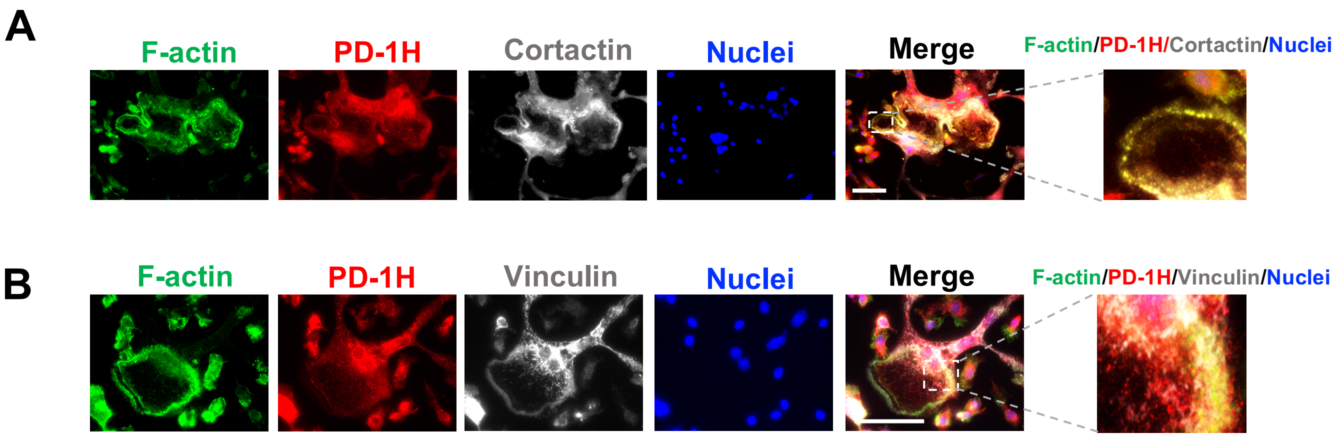

Supplemental Figure 3. PD-1H co-localizes with cytoskeletal proteins Cortactin and Vinculin at the sealing zone. Nonadherent bone marrow cells from WT mice were cultured for OCL differentiation and subjected to IF staining using Acti-stain 488 phalloidin (for F-actin) (Green), and antibodies against PD-1H (Red), Cortactin (A, Grey), Vinculin (B, Grey) and DAPI (Blue). Scale bar, 100 μm. The experiment was repeated three times with similar results.

Supplemental Figure 4

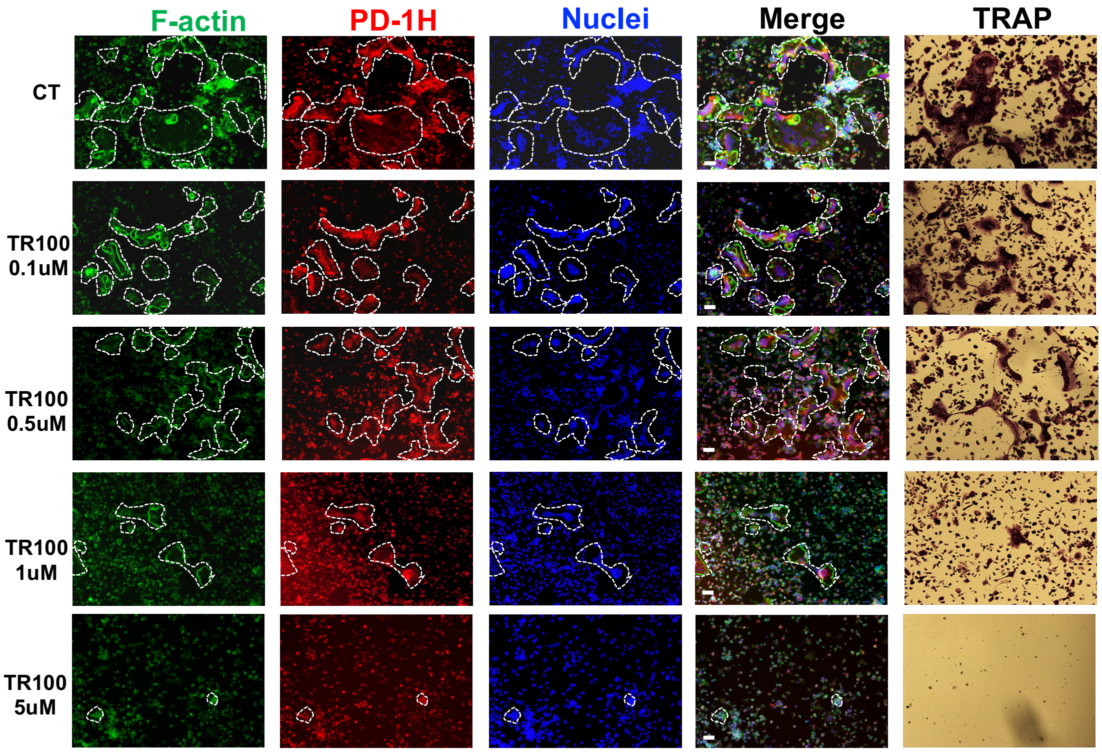

Supplemental Figure 4. F-actin assembly inhibitor TR100 disrupts OCL fusion. Nonadherent bone marrow cells from WT mice were cultured for OCL differentiation with different dosages of F-actin inhibitor TR100. On day 4, cells were fixed by 4%PFA and followed by IF staining or TRAP staining. Scale bar, 100 µm. The experiment was repeated three times with similar results.

Supplemental Figure 5

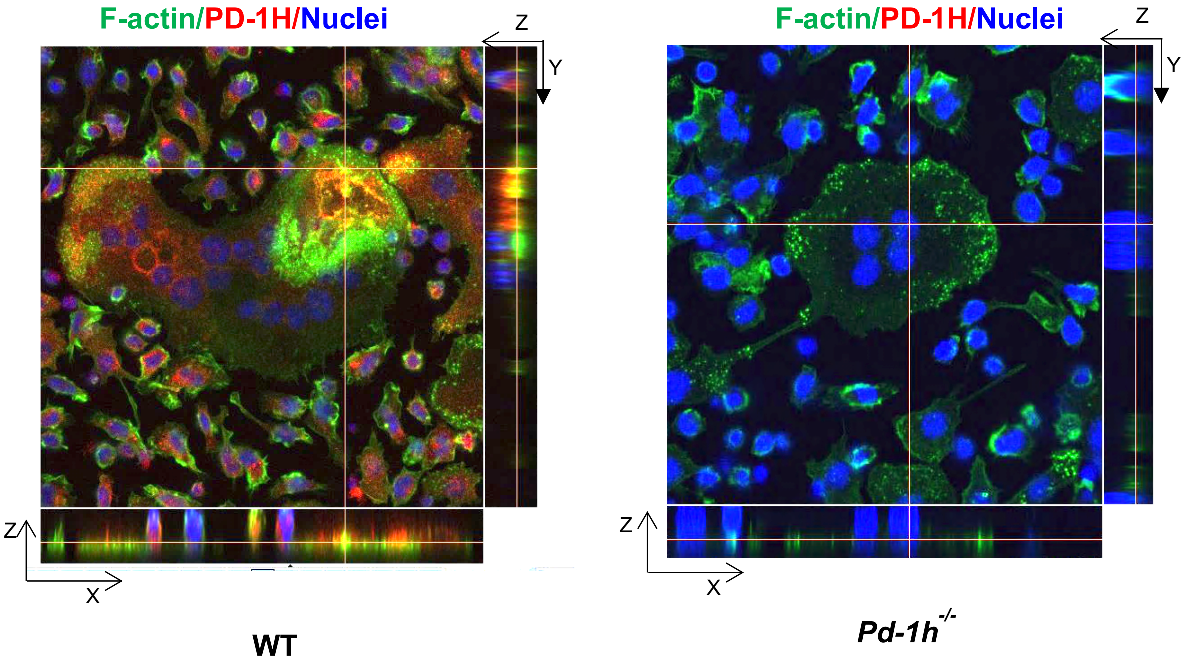

Supplemental Figure 5. 3-D dimensional localization of PD-1H and F-actin in OCL. BMCs of WT or *Pd-1h*$^{-/-}$ mice were cultured in OCL differentiation medium for 3 days and stained with Acti-stain 488 phalloidin (for F-actin) (green), PE-anti-PD-1H (red) and DAPI (blue). Images were obtained by confocal microscopy. X-Z and Y-Z planes of F-actin clusters and actin rings were analyzed by NIS Elements Software (NIKON).

Supplemental Figure 6

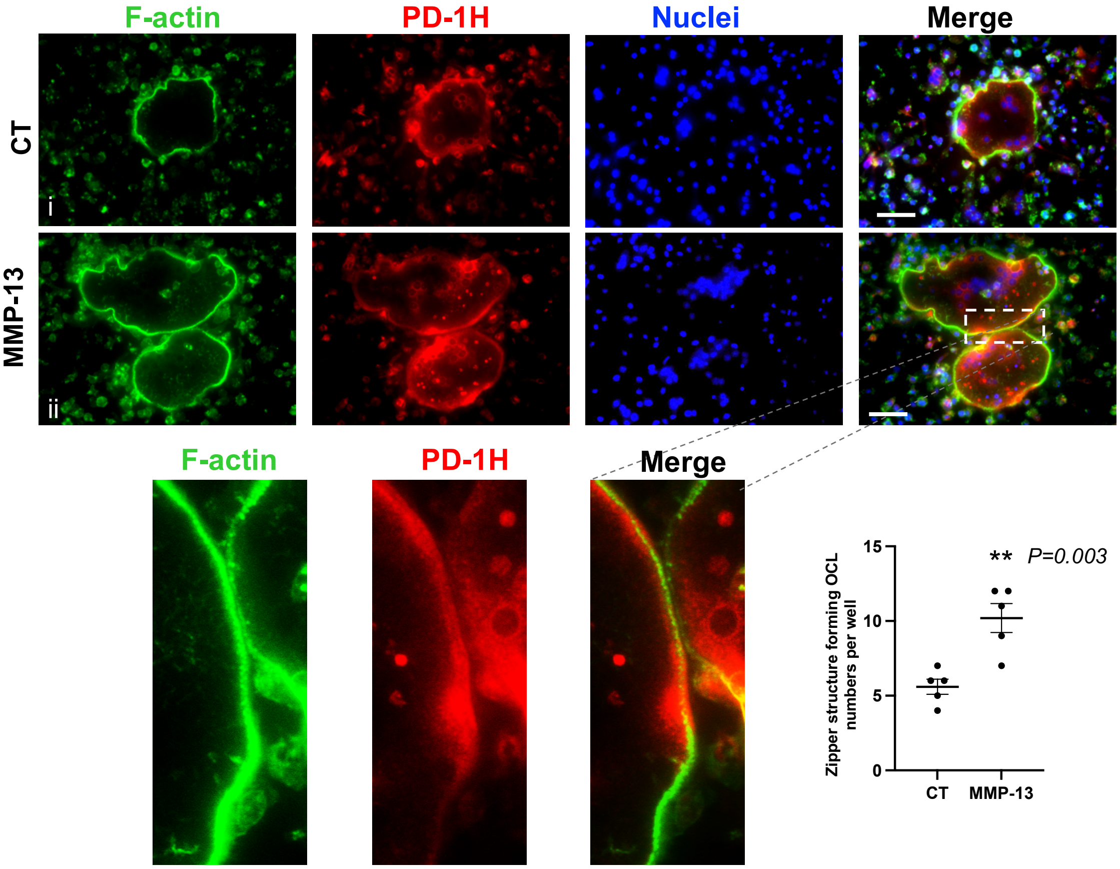

Supplemental Figure 6. PD-1H co-localizes with F-actin rings in zipper-like structures. BMCs of WT mice were cultured in OCL differentiation medium without or with MMP-13 and stained by Acti-stain 488 phalloidin (for F-actin) (green), PE-anti-PD-1H (red), and DAPI (blue). Scale bar, 100 µm. Zipper structure forming osteoclast number per well were counted (*n*=5 wells per group). Mean ± SEM. $^{**}P \leq 0.01$ by two-tailed Student's t-test.

Supplemental Figure 7

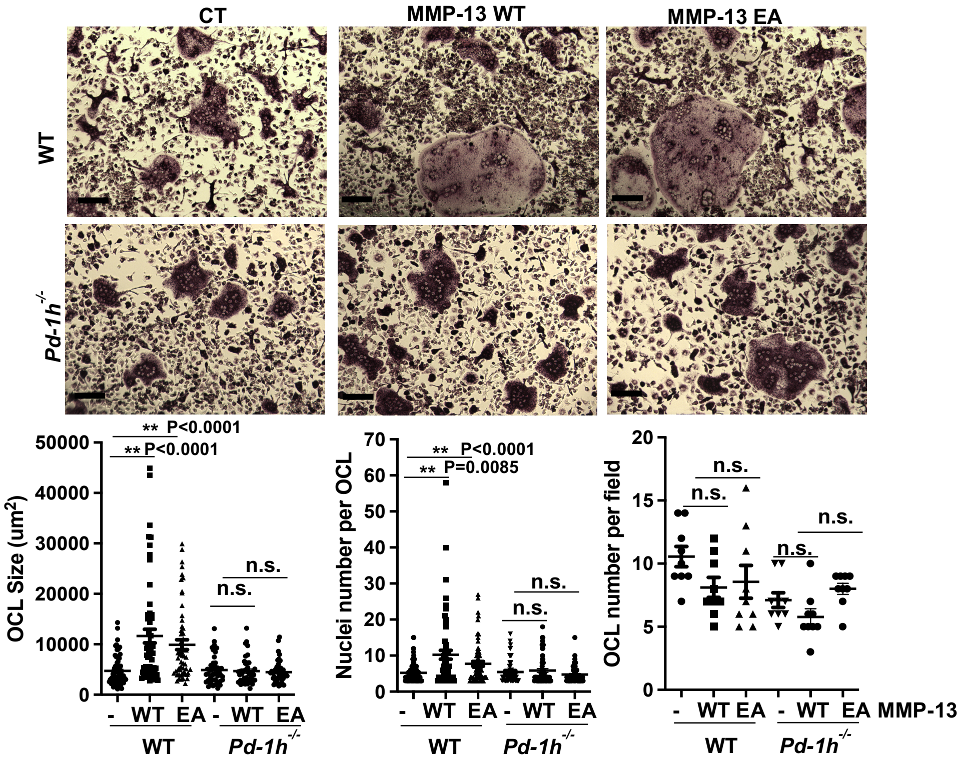

Supplemental Figure 7. MMP-13 proteolytic activity is not required for PD-1H mediated osteoclast activation. WT or *Pd-1h*$^{-/-}$ mice BMMNCs were cultured for osteoclast differentiation with either MMP-13 wild type zymogen or MMP-13 E223A enzymatic dead mutant, followed by TRAP staining. Nuclei number per OCL, average OCL size and OCL number per field were assessed by microscopy and ImageJ software. Nuclei number per OCL was measured based on the randomly imaged fields of OCLs. WT control *n*=87 cells; WT+MMP-13 WT *n*=64 cells; WT+MMP-13 EA *n*=71 cells; *Pd-1h*$^{-/-}$ CT *n*=59 cells; *Pd-1h*$^{-/-}$+ MMP-13 WT *n*=55 cells; *Pd-1h*$^{-/-}$+ MMP-13 EA *n*=61 cells. Average OCL size was measured based on the randomly imaged fields of OCLs: WT control *n*=73 cells; WT + MMP-13 WT *n*=57 cells; WT + MMP-13 EA *n*=57 cells; *Pd-1h*$^{-/-}$ CT *n*=43 cells; *Pd-1h*$^{-/-}$+ MMP-13 WT *n*=40 cells; *Pd-1h*$^{-/-}$+ MMP-13 EA n=51 cells. OCL number per field was assessed by *n*=10 randomly imaged fields. Mean ± SEM. $^{**}P \leq 0.01$ by one-way ANOVA.

Supplemental Figure 8

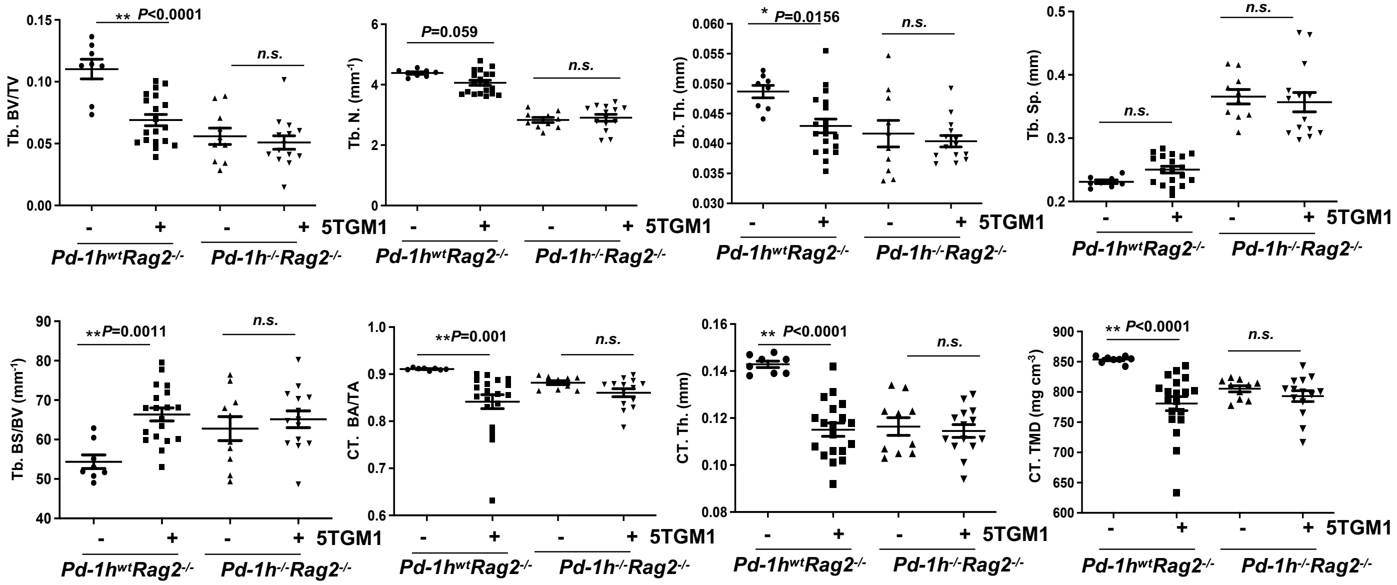

Supplemental Figure 8. *Pd-1h$^{-/-}$* in recipient mice blocked 5TGM1 MM induced bone loss. Microarchitectural parameters of the PBS or 5TGM1 injected *Pd-1h^{wt}Rag2$^{-/-}$* and *Pd-1h$^{-/-}$Rag2$^{-/-}$* mice were analyzed based on micro-qCT. Trabecular bone volume fraction (Tb. BV/TV), D.T. trabecular number (Tb. N.), D.T. trabecular thickness (Tb. Th.), D.T. trabecular spacing (Tb. Sp.), bone surface to bone volume ratio (Tb. BS/BV), average cross-sectional area of cortical bone (CT. BA/TA), cortical bone thickness (CT. Th.), and cortical tissue mineral density (CT. TMD) were analyzed. *Pd-1h^{wt}Rag2$^{-/-}$* control tibiae *n*=8; *Pd-1h^{wt}Rag2$^{-/-}$* 5TGM1 bearing tibiae *n*=19; *Pd-1h$^{-/-}$Rag2$^{-/-}$* control tibiae *n*=10; *Pd-1h$^{-/-}$Rag2$^{-/-}$* 5TGM1 bearing tibiae *n*=14. Results are presented as mean ± SEM. *$P \leq 0.05$, **$P \leq 0.01$* by one way ANOVA test.

Supplemental Figure 9

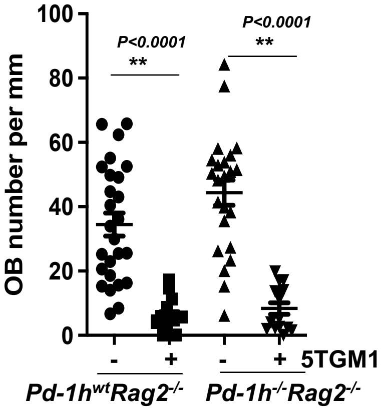

Supplemental Figure 9. *Pd-1h$^{-/-}$* in recipient mice did not affect 5TGM1 MM induced osteoblast inhibition. Decalcified tibiae were embedded in paraffin blocks followed by IHC staining for osteoblast using Osteocalcin antibody. Osteoblast number per mm of bone were assessed by microscopy based on the randomly imaged fields of bone and quantified by ImageJ. *Pd-1h^{wt}Rag2$^{-/-}$* control tibiae *n*=26 bone areas; *Pd-1h^{wt}Rag2$^{-/-}$* 5TGM1 bearing tibiae *n*=15 MM adjacent bone areas; *Pd-1h$^{-/-}$Rag2$^{-/-}$* control tibiae *n*=23 bone areas; *Pd-1h$^{-/-}$Rag2$^{-/-}$* 5TGM1 bearing tibiae *n*=14 MM adjacent bone areas. Results are presented as mean ± SEM.**$P \leq 0.01$ by one-way ANOVA.

Supplemental Figure 10

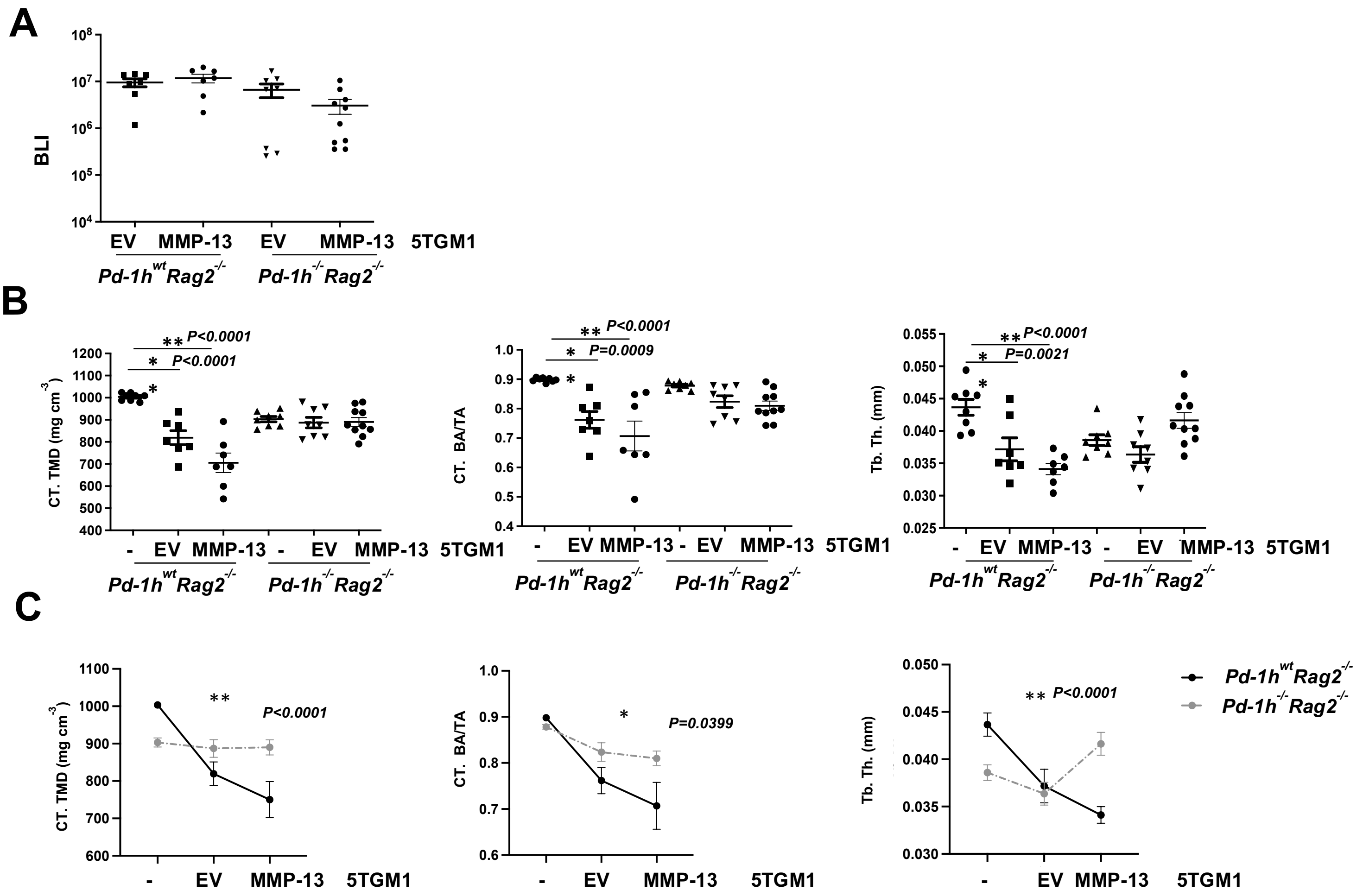

Supplemental Figure 10. ***Pd-1h*$^{-/-}$ in recipient mice blocked MMP-13 overexpressed 5TGM1 MM induced bone loss.** PBS, EV transduced 5TGM1-GFP-Luc or MMP-13 overexpressing 5TGM1-GFP-Luc cells were intratibially injected into *Pd-1h*^{wt}*Rag2*$^{-/-}$ and *Pd-1h*$^{-/-}$*Rag2*$^{-/-}$ mice (female, 13-16 weeks old). **(A)** 3 weeks later, tumor burden was detected by BLI. *Rag2*$^{-/-}$ 5TGM1 EV tumor bearing tibiae *n*=7; *Rag2*$^{-/-}$ 5TGM1 MMP-13 OE bearing tibiae *n*=7; *Pd-1h*$^{-/-}$*Rag2*$^{-/-}$ 5TGM1 EV bearing tibiae *n*=8; *Pd-1h*$^{-/-}$*Rag2*$^{-/-}$ 5TGM1 MMP-13 OE bearing tibiae *n*=10. Results are presented as mean ± SEM and analyzed by one-way ANOVA test. **(B)** Tibiae were then fixed in 10% formalin and analyzed by micro-CT scanning. Microarchitectural parameters were analyzed based on micro-qCT: cortical tissue mineral density (CT. TMD), average cross-sectional area of cortical bone (CT. BA/TA), D.T. trabecular thickness (Tb. Th.). *Pd-1h*^{wt}*Rag2*$^{-/-}$ control tibiae *n*=8; *Pd-1h*^{wt}*Rag2*$^{-/-}$ 5TGM1 EV tumor bearing tibiae *n*=7; *Pd-1h*^{wt}*Rag2*$^{-/-}$ 5TGM1 MMP-13 OE bearing tibiae *n*=7; *Pd-1h*$^{-/-}$*Rag2*$^{-/-}$ control tibiae *n*=8; *Pd-1h*$^{-/-}$*Rag2*$^{-/-}$ 5TGM1 EV bearing tibiae *n*=8; *Pd-1h*$^{-/-}$*Rag2*$^{-/-}$ 5TGM1 MMP-13 OE bearing tibiae *n*=10. Results are presented as mean ± SEM. *$P \leq 0.05$, **$P \leq 0.01$ by two-way ANOVA with Tukey's multiple comparisons. **(C)** Same data from (B) was presented by interaction curves. Results are presented as mean ± SEM. *$P \leq 0.05$, **$P \leq 0.01$ by two-way ANOVA.

Supplemental Figure 11

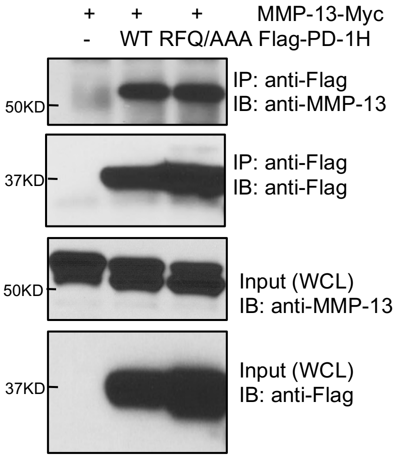

Supplemental Figure 11. PD-1H$^{R86A/F94A/Q95A}$ triple mutation does not affect MMP-13 binding. HEK293 cells were transfected with MMP-13 alone or together with Flag-PD-1H full length (FL) or R86A/F94A/Q95A triple point mutation (RFQ/AAA), followed by IP using Flag antibody. Whole cell lysates were analyzed as input. The experiment was repeated three times with similar results.

Supplemental Figure 12

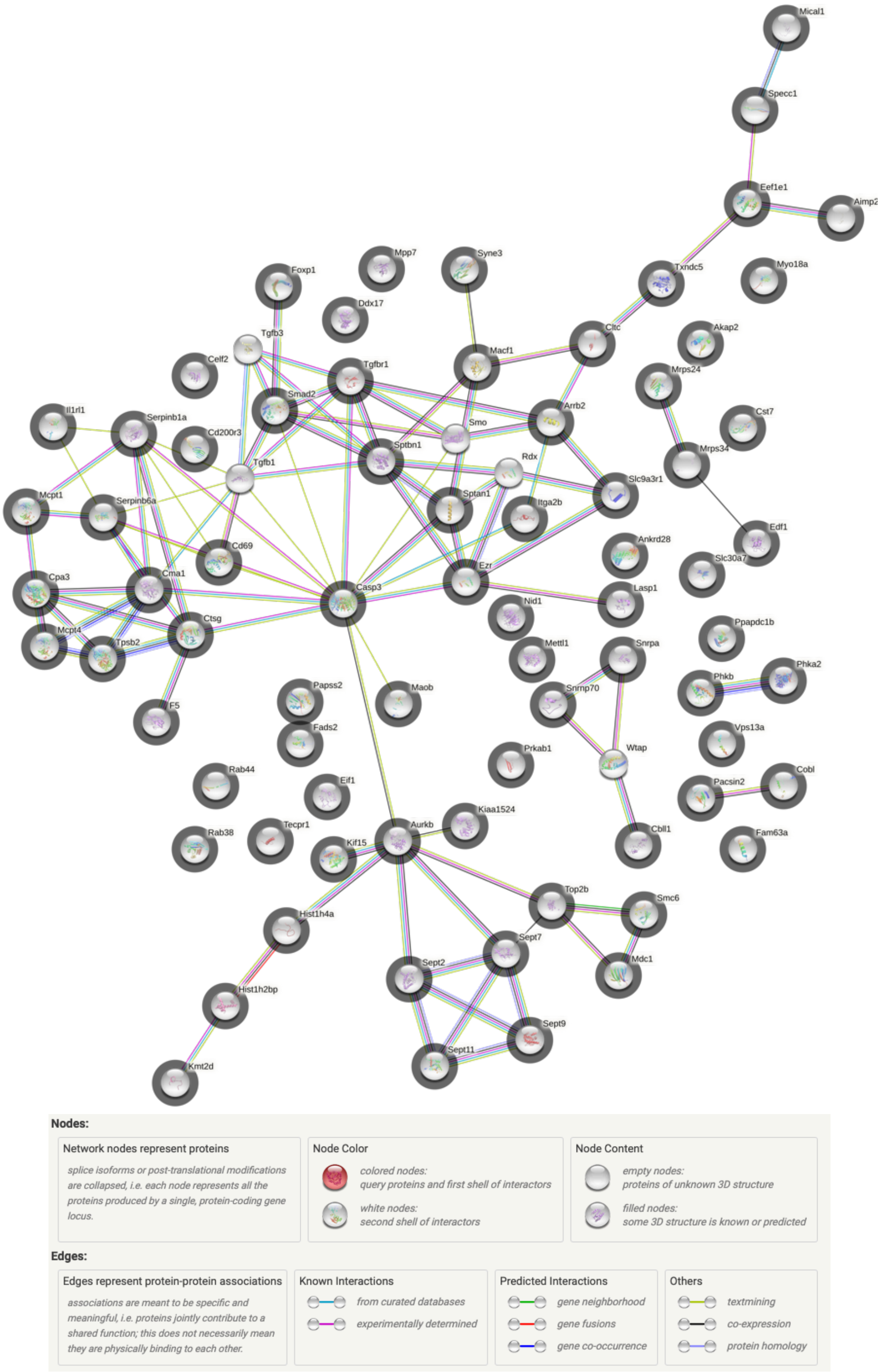

Supplemental Figure 12. PD-1H interaction network analyzed by STRING Database. The top 75 proteins enriched in PD-1H pull-down samples were analyzed by STRING Database.

Supplemental Figure 13

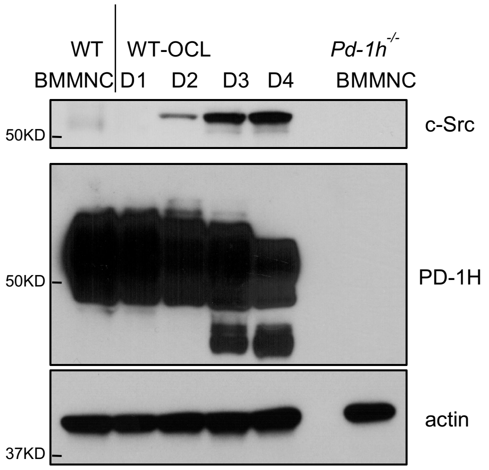

Supplemental Figure 13. c-Src expression is elevated during osteoclast differentiation. Bone marrow non-adherent mononuclear cells (BMMNC) from WT mice were cultured for osteoclast differentiation in the presence of M-CSF and RANKL and cell lysates were collected at the indicated time point for WB. BMMNC from *Pd-1h*$^{-/-}$ mice were loaded as control. The experiment was repeated three times with similar results.

Uncropped scan of blots

Supplemental Figure 1B

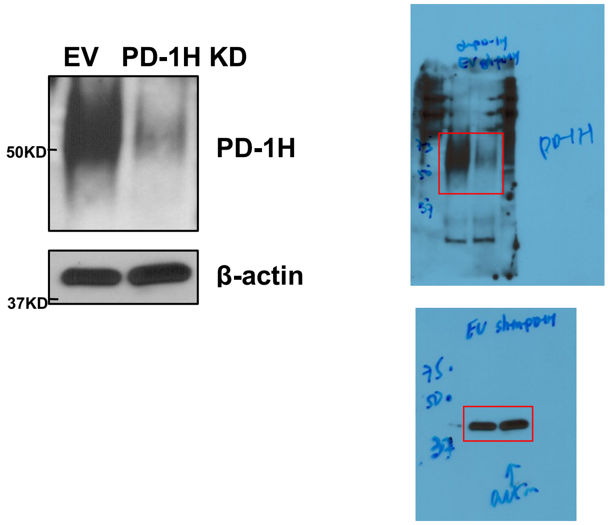

Supplemental Figure2A

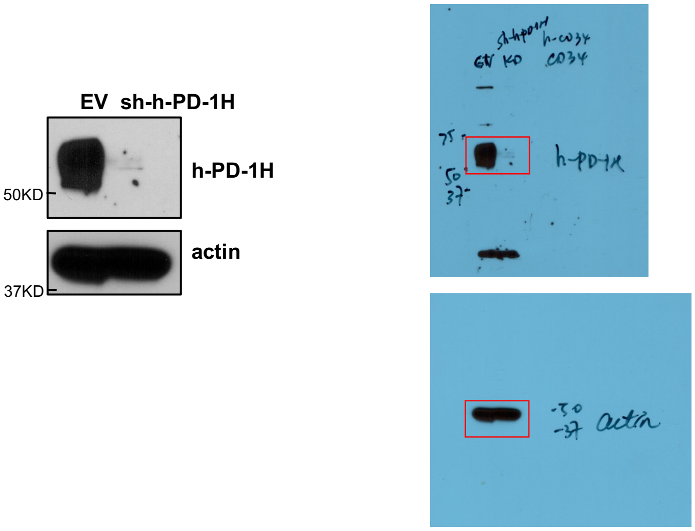

Supplemental Figure 2B

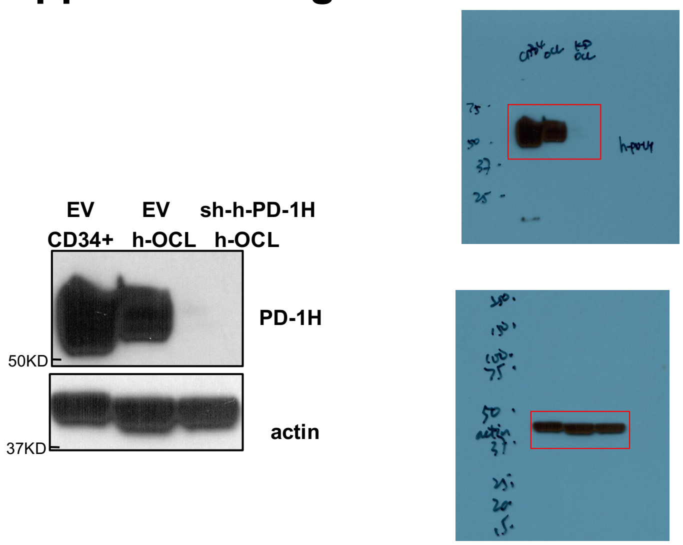

Supplemental Figure 11

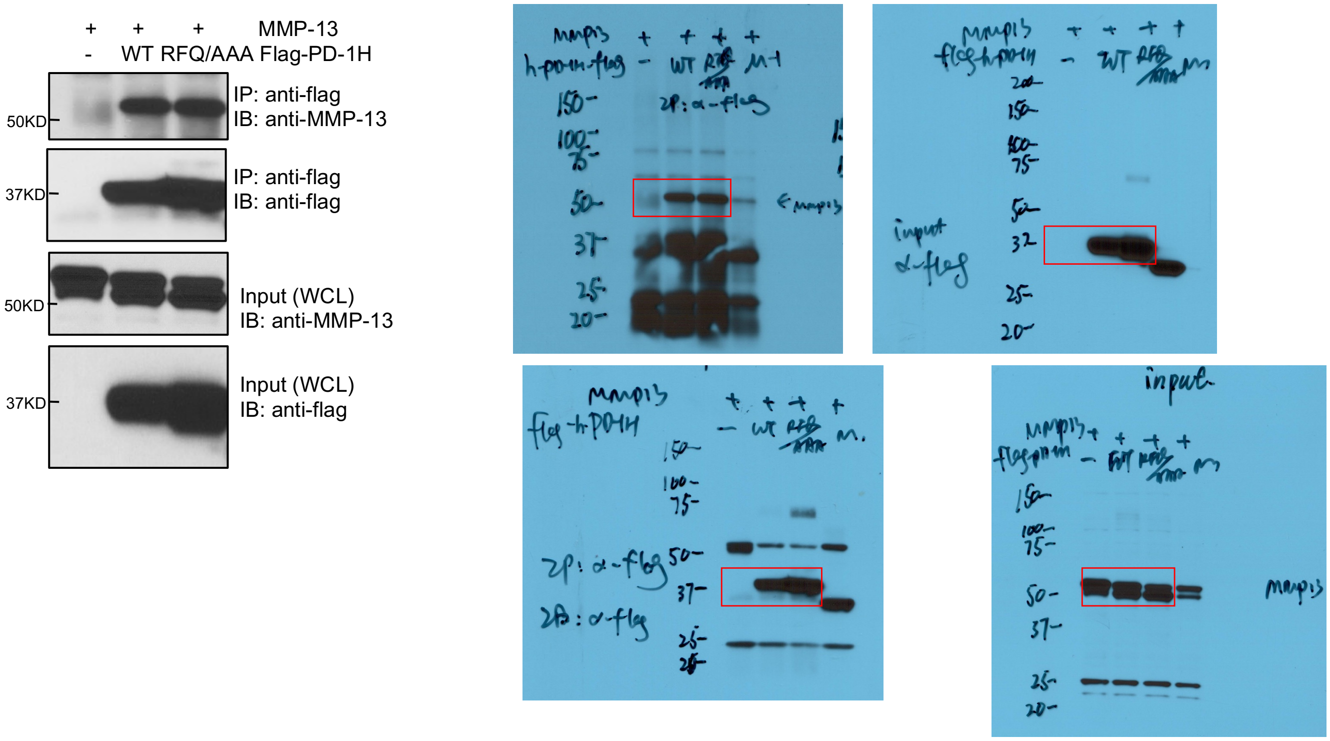

Supplemental Figure 13

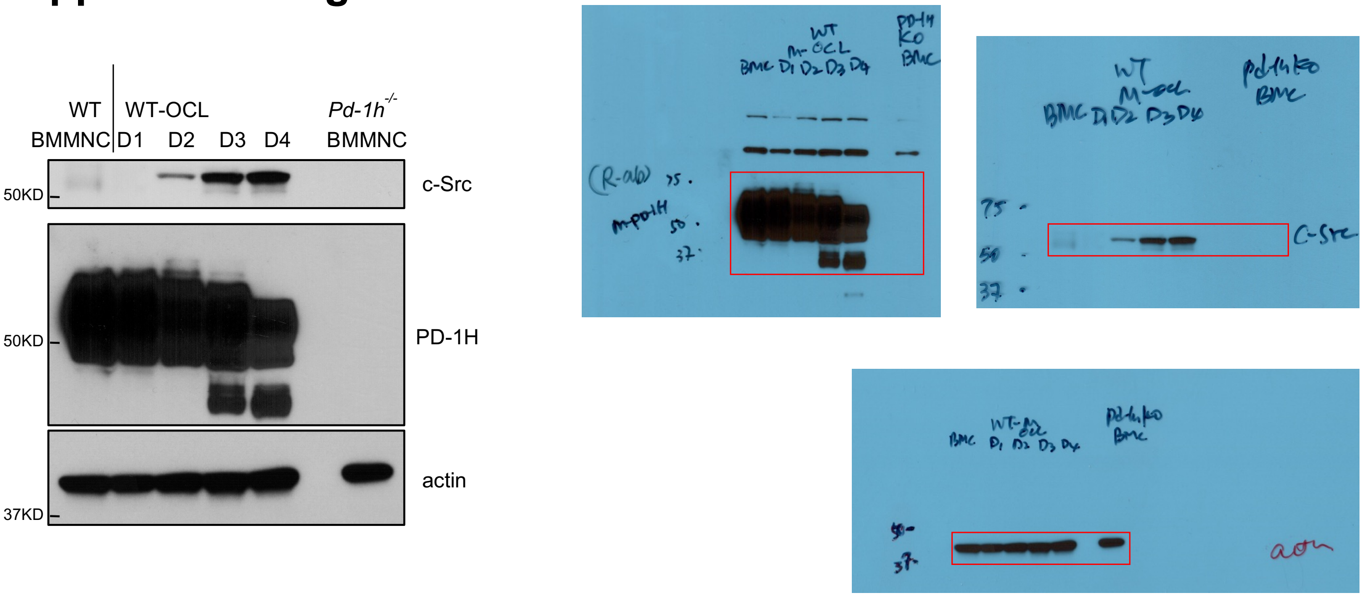

Supplement: Supplementary file 1 — Supplementary Information [file 41467_2023_39769_MOESM1_ESM.pdf]
