# Supplementary material for: The checkpoint inhibitor PD-1H/VISTA controls osteoclast-mediated multiple myeloma bone disease
Source: Nat Commun. 2023 Jul 17;14:4271. doi: 10.1038/s41467-023-39769-8 (PMC10352288; doi:10.1038/s41467-023-39769-8)
Supplement: Supplementary file 2 — Description of Additional Supplementary File [file 41467_2023_39769_MOESM2_ESM.pdf]

Description of Additional Supplementary Files

Supplementary Data 1. MMP-13-His6 pulldown data collected on the Synapt G2 HDMS mass spectrometer.

Supplementary Data 2. List of 28 peptides that have been identified from the bait pro-MMP-13 protein by MS.

Supplementary Data 3. PD-1H-His6 pulldown data collected on the Q Exactive HF mass spectrometer.
