# Supplementary material for: The checkpoint inhibitor PD-1H/VISTA controls osteoclast-mediated multiple myeloma bone disease
Source: Nat Commun. 2023 Jul 17;14:4271. doi: 10.1038/s41467-023-39769-8 (PMC10352288; doi:10.1038/s41467-023-39769-8)
Supplement: Supplementary file 6 — Reporting Summary [file 41467_2023_39769_MOESM6_ESM.pdf]

nature portfolio

Corresponding author(s): Suzanne Lentzsch
Last updated by author(s): May 17, 2023

Reporting Summary

Nature Portfolio wishes to improve the reproducibility of the work that we publish. This form provides structure for consistency and transparency in reporting. For further information on Nature Portfolio policies, see our Editorial Policies and the Editorial Policy Checklist.

Statistics

For all statistical analyses, confirm that the following items are present in the figure legend, table legend, main text, or Methods section.

n/a	Confirmed	
☐	☒	The exact sample size (*n*) for each experimental group/condition, given as a discrete number and unit of measurement
☐	☒	A statement on whether measurements were taken from distinct samples or whether the same sample was measured repeatedly
☐	☒	The statistical test(s) used AND whether they are one- or two-sided<br>*Only common tests should be described solely by name; describe more complex techniques in the Methods section.*
☐	☒	A description of all covariates tested
☐	☒	A description of any assumptions or corrections, such as tests of normality and adjustment for multiple comparisons
☐	☒	A full description of the statistical parameters including central tendency (e.g. means) or other basic estimates (e.g. regression coefficient) AND variation (e.g. standard deviation) or associated estimates of uncertainty (e.g. confidence intervals)
☐	☒	For null hypothesis testing, the test statistic (e.g. *F*, *t*, *r*) with confidence intervals, effect sizes, degrees of freedom and *P* value noted<br>*Give P values as exact values whenever suitable.*
☒	☐	For Bayesian analysis, information on the choice of priors and Markov chain Monte Carlo settings
☒	☐	For hierarchical and complex designs, identification of the appropriate level for tests and full reporting of outcomes
☒	☐	Estimates of effect sizes (e.g. Cohen's *d*, Pearson's *r*), indicating how they were calculated

Our web collection on statistics for biologists contains articles on many of the points above.

Software and code

Policy information about availability of computer code

Data collection	Leica AF6000, NIS Elements Software (NIKON), VivaCT40 (Scanco Medical)
Data analysis	Protein Lynx Global Server (Vers.2.5, RC9) (Waters Corp.), Rosetta Elucidator software V. 4.0.0.2. 31) (Ceiba Solutions, Inc.), Mascot and Elucidator software, ImageJ, Excel (version 16.66.1) and Graphpad Prism 9.

For manuscripts utilizing custom algorithms or software that are central to the research but not yet described in published literature, software must be made available to editors and reviewers. We strongly encourage code deposition in a community repository (e.g. GitHub). See the Nature Portfolio guidelines for submitting code & software for further information.

Data

Policy information about availability of data

All manuscripts must include a data availability statement. This statement should provide the following information, where applicable:

- Accession codes, unique identifiers, or web links for publicly available datasets
- A description of any restrictions on data availability
- For clinical datasets or third party data, please ensure that the statement adheres to our policy

All raw mass spectrometry files produced in this work are publicly available at the MassIVE proteomics repository under accession MSV000089119: (https://massive.ucsd.edu/ProteoSAFe/dataset.jsp?accession=MSV000089119) (doi:10.25345/C5251FP6G). All data generated or analyzed during this study are included within the article and its Supplementary Information files. Source data are provided with this paper.

Research involving human participants, their data, or biological material

Policy information about studies with human participants or human data. See also policy information about sex, gender (identity/presentation), and sexual orientation and race, ethnicity and racism.

Reporting on sex and gender	N/A
Reporting on race, ethnicity, or other socially relevant groupings	N/A
Population characteristics	N/A
Recruitment	N/A
Ethics oversight	N/A

Note that full information on the approval of the study protocol must also be provided in the manuscript.

Field-specific reporting

Please select the one below that is the best fit for your research. If you are not sure, read the appropriate sections before making your selection.

☒ Life sciences ☐ Behavioural & social sciences ☐ Ecological, evolutionary & environmental sciences

For a reference copy of the document with all sections, see nature.com/documents/nr-reporting-summary-flat.pdf

Life sciences study design

All studies must disclose on these points even when the disclosure is negative.

Sample size	The sample size was estimated based on the pilot experiments and previously published data (doi:10.1172/JCI80276) so that appropriate tests would yield significant results. The exact N numbers are indicated in figure legends.
Data exclusions	No data were excluded from the study.
Replication	The pull-down and mass spectrum assay was conducted once using 3 replicated samples, and each sample was analyzed twice. All the other in vitro experiments were repeated at least 3 times. All attempts at replication were successful.
Randomization	For in vivo studies, all mice were randomly allocated to 5TGM1 MM (EV or MMP-13 OE) or PBS injection. For in vitro cell experiments, all cells in each experiment were randomly aliquoted from the same parental cells.
Blinding	Investigators who conducted western blotting and Q-PCR assays were blinded to sample treatment. In mice MM bone disease studies, the investigators conducted bone micro-CT analysis and immunohistology staining using a sample number and were blinded to the genotype and treatment. In vitro osteoclast assays were not blinded to investigators since the experiments were not applicable for blinding.

Reporting for specific materials, systems and methods

We require information from authors about some types of materials, experimental systems and methods used in many studies. Here, indicate whether each material, system or method listed is relevant to your study. If you are not sure if a list item applies to your research, read the appropriate section before selecting a response.

Materials & experimental systems

n/a	Involved in the study	
☐	☒	Antibodies
☐	☒	Eukaryotic cell lines
☒	☐	Palaeontology and archaeology
☐	☒	Animals and other organisms
☒	☐	Clinical data
☒	☐	Dual use research of concern
☒	☐	Plants

Methods

n/a	Involved in the study	
☒	☐	ChIP-seq
☒	☐	Flow cytometry
☒	☐	MRI-based neuroimaging

Antibodies

Antibodies used	PE-anti-m-PD-1H for IF (Biolegend, Cat# 150204)<br>Alexa Fluor647-anti-cortactin for IF (Abcam, Cat#ab202650)<br>Alexa Fluor647-anti-vinculin for IF (Abcam, Cat# ab196579)<br>Rabbit anti-c-Src (36D10) for IF (CST, Cat# 2109)<br>Mouse anti-Rac1 for IF (Thermo Fisher Scientific, Cat# 16118)<br>Alexa Fluor 488-conjugated goat anti-rabbit (Invitrogen, Cat# A-11034)<br>Alexa Fluor 488-conjugated donkey anti-mouse (Invitrogen, Cat# A-21202)<br>Rabbit anti-MMP-13 for WB (Abcam, Cat# 39012)<br>Sheep anti-m-PD-1H for WB (R&D Systems, Cat# AF7005)<br>Sheep anti-h-PD-1H for WB (R&D Systems, Cat# AF7126)<br>Mouse anti-DC-STAMP for WB (clone 1A2), (EMD Millipore, Cat# MABF39-I)<br>Mouse anti-NFATc1 (7A6) for WB (Santa Cruz Biotechnology, Cat# sc-7294)<br>Rabbit anti-P-ERK1/2 (D13.14.4E) for WB (CST, Cat# 4370)<br>Rabbit anti-ERK1/2 for WB (CST, Cat# 9102)<br>Rabbit anti-P-c-Src (Y416) for WB (CST, Cat# 2101)<br>Rabbit anti-c-Src (3266) for WB (CST, Cat# 2123)<br>Rabbit anti-h/m-PD-1H (D5L5T) for IP and WB (CST, Cat# 54979)<br>Rabbit anti-osteocalcin for IHC (Abcam, Cat# 93876)<br>anti-Flag tag for IP and WB (Cat# 3165, Sigma Aldrich)<br>anti-c-myc(9E10) tag for IP and WB (Cat#sc-40, Santa Cruz Biotechnology)<br>Mouse anti-b-actin (Sigma-Aldrich, Cat# A5441)
Validation	Validation of antibodies can be found on their corresponding manufacturer websites. Abcam antibodies has knock-out validation via CRISPR-Cas genome-deiting; Biolegend antibodies are quality control tested by IF staining.<br>In our experiments, the specificities of the PD-1H antibodies: PE-anti-m-PD-1H for IF (Biolegend, Cat# 150204), Sheep anti-m-PD-1H for WB (R&D Systems, Cat# AF7005), Sheep anti-h-PD-1H for WB (R&D Systems, Cat# AF7126) and Rabbit anti-h/m-PD-1H (D5L5T) for IP and WB (CST, Cat# 54979) were validated by western blotting or IF staining on wild type vs Pd-1h knockout or knockdown cells as shown in the applicable figures.<br>Tag antibodies: anti-Flag tag (Cat# 3165, Sigma Aldrich); anti-c-myc tag (Cat#sc-40, Santa Cruz Biotechnology) were validated by WB comparing tag expressing cell lysates to non-transfected controls.<br>The other antibodies Mouse anti-DC-STAMP for WB (clone 1A2) (EMD Millipore, Cat# MABF39-I), Mouse anti-NFATc1 (7A6) for WB (Santa Cruz Biotechnology, Cat# sc-7294), Rabbit anti-P-ERK1/2 (D13.14.4E) for WB (CST, Cat# 4370), Rabbit anti-ERK1/2 for WB (CST, Cat# 9102), Rabbit anti-P-c-Src (Y416) for WB (CST, Cat# 2101), Rabbit anti-c-Src (3266) for WB (CST, Cat# 2123) and Mouse anti-b-actin (Sigma-Aldrich, Cat# A5441) have been confirmed by WB detection of bands at the predicted size.

Eukaryotic cell lines

Policy information about cell lines and Sex and Gender in Research

Cell line source(s)	HEK293T cells were purchase from Takara.<br>RAW264.7 cells were purchased from ATCC.<br>5TGM1 cells were kindly provided by Dr. Roodman from Indiana University.
Authentication	All cell lines have been authenticated by short tandem repeat (STR) profiling.
Mycoplasma contamination	All cell lines were tested negative for Myoplamsa contamination.
Commonly misidentified lines<br>(See ICLAC register)	No misidentified cell line was used in this study.

Animals and other research organisms

Policy information about studies involving animals; ARRIVE guidelines recommended for reporting animal research, and Sex and Gender in Research

Laboratory animals	C57BL/6 WT, Rag2-/-, Pd-1h-/- and Rag2-/-Pd-1h-/- mice. All experimental animals were used at 8-16 weeks of age as indicated in the individual experiments. Sex of the experimental mice are indicated in the individual experiments.Mice were housed with food (PicoLab Rodent Diet 20, Cat# 5053) and water ad libitum in a temperature and humidity-controlled environment on a 12-h light–dark cycle.
Wild animals	This study does not involve wild animals.
Reporting on sex	Animal experiment #1 was conducted on male mice and #2 was conducted on female mice. Sex information has been included in the legend and data was analyzed within each individual sex group.
Field-collected samples	This study does not involve field-collected samples.
Ethics oversight	The reported research complies with all relevant ethical regulations. All animal procedures were reviewed and approved by the IACUC of Columbia University, New York (Protocols AC-AAAE9803 and AC-AAAW6454).

Note that full information on the approval of the study protocol must also be provided in the manuscript.
